# Inference on the stress strength reliability with exponentiated generalized Marshall Olkin-G distribution

**DOI:** 10.1371/journal.pone.0280183

**Published:** 2023-08-09

**Authors:** Neama Salah Youssef Temraz

**Affiliations:** 1 Prince Sattam Bin Abdulaziz University, College of Arts and Sciences, Al-Kharj, KSA; 2 Faculty of Science, Mathematics Department, Tanta University, Tanta, Egypt; University of Eastern Finland, FINLAND

## Abstract

In this paper, an inference on stress-strength reliability model is introduced in case of the exponentiated generalized Marshall Olkin G family of distributions. The maximum likelihood estimator of the stress-strength reliability function is deduced. An asymptotic confidence and bootstrap confidence intervals for the stress-strength reliability function are derived. A Bayesian inference is introduced for the stress-strength reliability. A simulation is introduced to obtain the maximum likelihood and Bayesian estimates for the stress strength reliability. Real data applications are provided to show the results for the stress-strength model and compare the exponentiated generalized Marshall Olkin-G distribution with other distributions.

## 1. Introduction

In life testing, it is very important to study the stress-strength reliability which refers to the quantity *R* = *P* (*Y* < *X*) and it is assumed that two independent random variables *X* and *Y* where *X* represents the stress of a component and *Y* represents the strength of the component such that the component fails in case that the stress exceeds the strength.

Al-Mutairi et. al [1] presented an inference on stress-strength reliability from Lindley distribution. Rao et. al [2] introduced an estimation of stress–strength reliability from inverse Rayleigh distribution. Singh et al. [3] presented an estimation of the stress strength reliability subject to the inverted exponential distribution. Al-Mutairi [4] discussed an inference on stress-strength reliability from weighted Lindley distributions. Sharma [5] presented a stress-strength reliability model subject to the inverse Lindley distribution with application to head and neck cancer data.

Mokhlis et al. [6] proposed the stress-strength reliability with general form distributions. Çetinkaya and Genç [7] introduced the stress–strength reliability estimation under the standard two-sided power distribution. Almarashi [8] introduced an estimation of the stress-strength reliability for Weibull distribution with application. Muhammad et al. [9] proposed an estimation of the reliability of a stress–strength system from Poisson half logistic distribution. Nojosa and Rathie [10] introduced Stress–strength reliability estimation involving generalized gamma and Weibull distributions.

Alamri et al. [11] studied the stress-strength reliability where the strength (*X*) follows Rayleigh-half-normal distribution and stress follows Rayleigh-half-normal distribution, exponential distribution, Rayleigh distribution, and half-normal distribution, respectively. Hassan et al. [12] introduced stress–strength reliability for the generalized inverted exponential distribution using median ranked set sampling. Abu El Azm et al. [13] presented a study for stress-strength reliability subject to exponentiated inverted Weibull distribution with application on breaking of jute fiber and carbon fibers. Jha et al. [14] discussed the multicomponent stress-strength reliability estimation based on unit generalized Rayleigh distribution.

Jha et al. [15] proposed the multicomponent stress-strength reliability estimation based on unit generalized exponential distribution. Maurya et al. [16] introduced a reliability estimation in a multicomponent stress-strength model based on inverse Weibull distribution. Jovanovic et al. [17] proposed an inference on reliability of stress-strength model with Peng-Yan extended Weibull distributions. Sabry et al. [18] presented a Monte Carlo simulation of the stress-strength model and reliability estimation for extension of the exponential distribution. Zarei and Shahrestani [19] proposed the Bayes and empirical Bayes estimator of reliability function in multicomponent stress-strength system based on generalized Rayleigh distribution.

The exponentiated Marshall Olkin family distribution is used in practice since it makes the kurtosis of data more flexible compared to the baseline model and is used to construct heavy-tailed distributions for modeling real data. Special models can be constructed from the exponentiated Marshall Olkin family distribution with all types of the hazard rate functions. The exponentiated Marshall Olkin family distribution is used to provide consistently better fits than other generated models under the same baseline distribution. The Marshall Olkin family distribution is introduced and discussed in many papers in literature such as [20–26].

This paper deals with the inference of the stress-strength reliability in case of the stress and strength variables follow the exponentiated generalized Marshall Olkin G family of distributions. The maximum likelihood estimation of the stress-strength reliability is discussed. An asymptotic confidence and bootstrap confidence intervals for the stress-strength reliability function are discussed. Bayesian inference and the credible interval for the stress-strength reliability function are introduced. A simulation is carried out to obtain the results for the stress strength reliability in case of the exponentiated generalized Marshall Olkin Weibull distribution. Real data applications are introduced to study the goodness of fit of the real datasets.

## 2. Exponentiated generalized Marshall Olkin-G distribution

Handique et al. [20] introduced the exponentiated generalized Marshall Olkin G family of distributions (EGMO-G) with cumulative distribution function and probability density function defined as follows

$$F(x)={\left\{1-{\left[\frac{\vartheta \bar{G}(x;\lambda_{k})}{1-\bar{\vartheta }\bar{G}(x;\lambda_{k})}\right]}^{a}\right\}}^{b},\;x>0,a>0,b>0,\vartheta >0,\lambda_{k}>0$$

and

$$f(x)=\frac{ab\vartheta^{a}g(x;\lambda_{k}){\left[\bar{G}(x;\lambda_{k})\right]}^{a-1}}{{\left[1-\bar{\vartheta }\bar{G}(x;\lambda_{k})\right]}^{a+1}}{\left\{1-{\left[\frac{\vartheta \bar{G}(x;\lambda_{k})}{1-\bar{\vartheta }\bar{G}(x;\lambda_{k})}\right]}^{a}\right\}}^{b-1}$$

where *G*(*x*; *λ*_*k*_) and *g*(*x*; *λ*_*k*_) are the cumulative distribution function and the probability density function of a random variable *X*, respectively, with parameter vector *λ*_*k*_, *k* = 1,2,…,*l*.

For example, the cumulative distribution function and the probability density function of a random variable *X* that follows the exponentiated generalized Marshall Olkin Weibull distribution (EGMO-W) are given by

F(x)={1−[ϑe−(xβ)γ1−ϑ¯e−(xβ)γ]a}b,x>0,a>0,b>0,ϑ>0,γ>0,β>0

and

f(x)=abϑa(γβ)(xβ)γ−1e−(xβ)γ[e−(xβ)γ]a−1[1−ϑ¯e−(xβ)γ]a+1{1−[ϑe−(xβ)γ1−ϑ¯e−(xβ)γ]a}b−1

where *a*, *b*, *ϑ* and *γ* are the shape parameters and *β* is the scale parameter.

And the cumulative distribution function and the probability density function of a random variable *X* that follows the exponentiated generalized Marshall Olkin exponential distribution (EGMO-E) are given by

$$F(x)={\left\{1-{\left[\frac{\vartheta e^{-\lambda x}}{1-\bar{\vartheta }e^{-\lambda x}}\right]}^{a}\right\}}^{b},\;x>0,a>0,b>0,\vartheta >0,\lambda >0$$

and

$$f(x)=\frac{ab\vartheta^{a}\lambda e^{-\lambda x}{\left[e^{-\lambda x}\right]}^{a-1}}{{\left[1-\bar{\vartheta }e^{-\lambda x}\right]}^{a+1}}{\left\{1-{\left[\frac{\vartheta e^{-\lambda x}}{1-\bar{\vartheta }e^{-\lambda x}}\right]}^{a}\right\}}^{b-1}$$

where *a*, *b*, *ϑ* and *γ* are the shape parameters and *λ* is the scale parameter.

## 3. Stress-strength reliability

Let X and Y are two independent random variables follow the EGMO-G distribution, then the stress-strength reliability function will be given by

$$R=P(Y<X)=\int_{0}^{\infty }\int_{0}^{x}f(x)f(y)dydx=\int_{0}^{\infty }[\int_{0}^{x}f_{y}(y)dy]f_{x}(x)dx=\int_{0}^{\infty }F_{y}(x)f_{x}(x)dx$$

$$R=\int_{0}^{\infty }{\left\{1-{\left[\frac{\vartheta \bar{G}(x;\lambda_{k})}{1-\bar{\vartheta }\bar{G}(x;\lambda_{k})}\right]}^{a}\right\}}^{b_{2}}\frac{ab_{1}\vartheta^{a}g(x;\lambda_{k}){\left[\bar{G}(x;\lambda_{k})\right]}^{a-1}}{{\left[1-\bar{\vartheta }\bar{G}(x;\lambda_{k})\right]}^{a+1}}{\left\{1-{\left[\frac{\vartheta \bar{G}(x;\lambda_{k})}{1-\bar{\vartheta }\bar{G}(x;\lambda_{k})}\right]}^{a}\right\}}^{b_{1}-1}dx$$

Solving this integral, an expression for the stress-strength reliability is obtained as

$$R=\frac{b_{1}}{b_{1}+b_{2}}$$ (1)

## 4. Maximum likelihood estimation

Assuming that the two independent random samples (*X*_1_, *X*_2_,…,*X*_*n*_) and (*Y*_1_, *Y*_2_,…,*Y*_*m*_) are selected from the EGMO-G distribution with the parameters (*b*_1_, *a*, *ϑ*, *λ*_*k*_) and (*b*_2_, *a*, *ϑ*, *λ*_*k*_), respectively. The likelihood function is given as follows

$$L=\prod_{i=1}^{n}f(x_{i})\prod_{j=1}^{m}f(y_{j})=\prod_{i=1}^{n}\frac{ab_{1}\vartheta^{a}g(x_{i};\lambda_{k}){\left[\bar{G}(x_{i};\lambda_{k})\right]}^{a-1}}{{\left[1-\bar{\vartheta }\bar{G}(x_{i};\lambda_{k})\right]}^{a+1}}{\left\{1-{\left[\frac{\vartheta \bar{G}(x_{i};\lambda_{k})}{1-\bar{\vartheta }\bar{G}(x_{i};\lambda_{k})}\right]}^{a}\right\}}^{b_{1}-1}$$

$$\prod_{j=1}^{m}\frac{ab_{2}\vartheta^{a}g(y_{j};\lambda_{k}){\left[\bar{G}(y_{j};\lambda_{k})\right]}^{a-1}}{{\left[1-\bar{\vartheta }\bar{G}(y_{j};\lambda_{k})\right]}^{a+1}}{\left\{1-{\left[\frac{\vartheta \bar{G}(y_{j};\lambda_{k})}{1-\bar{\vartheta }\bar{G}(y_{j};\lambda_{k})}\right]}^{a}\right\}}^{b_{2}-1}$$ (2)

The log-likelihood function is obtained as follows

$$logL=(n+m)\log\left(a\right)+a(n+m)\log\left(\vartheta \right)+n\log\left(b_{1}\right)+m\log\left(b_{2}\right)+\sum_{i=1}^{n}\log\left[g(x_{i};\lambda_{k})\right]+(a-1)\sum_{i=1}^{n}\log\left[\bar{G}(x_{i};\lambda_{k})\right]-(a+1)\sum_{i=1}^{n}\log\left[1-\bar{\vartheta }\bar{G}(x_{i};\lambda_{k})\right]+(b_{1}-1)\sum_{i=1}^{n}\log\left\{1-{\left[\frac{\vartheta \bar{G}(x_{i};\lambda_{k})}{1-\bar{\vartheta }\bar{G}(x_{i};\lambda_{k})}\right]}^{a}\right\}+\sum_{j=1}^{m}\log\left[g(y_{j};\lambda_{k})\right]+(a-1)\sum_{j=1}^{m}\log\left[\bar{G}(y_{j};\lambda_{k})\right]-(a+1)\sum_{j=1}^{m}\log\left[1-\bar{\vartheta }\bar{G}(y_{j};\lambda_{k})\right]+(b_{2}-1)\sum_{j=1}^{m}\log\left\{1-{\left[\frac{\vartheta \bar{G}(y_{j};\lambda_{k})}{1-\bar{\vartheta }\bar{G}(y_{j};\lambda_{k}]}\right)}^{a}\right\}$$

The partial derivatives of the log-likelihood function with respect to (*b*_1_, *b*_2_, *ϑ*, *a*, *λ*_*k*_) are obtained as follows

$$\frac{\partial \log L}{\partial b_{1}}=\frac{n}{b_{1}}+\sum_{i=1}^{n}\log\left\{1-{\left[\frac{\vartheta \bar{G}(x_{i};\lambda_{k})}{1-\bar{\vartheta }\bar{G}(x_{i};\lambda_{k})}\right]}^{a}\right\}$$ (3)

$$\frac{\partial \log L}{\partial b_{2}}=\frac{m}{b_{2}}+\sum_{j=1}^{m}\log\left\{1-{\left[\frac{\vartheta \bar{G}(y_{j};\lambda_{k})}{1-\bar{\vartheta }\bar{G}(y_{j};\lambda_{k})}\right]}^{a}\right\}$$ (4)

$$\frac{\partial logL}{\partial \vartheta }=\frac{a(n+m)}{\vartheta }-(a+1)\sum_{i=1}^{n}\frac{\vartheta \bar{G}(x_{i};\lambda_{k})}{1-\bar{\vartheta }\bar{G}(x_{i};\lambda_{k})}+(b_{1}-1)\sum_{i=1}^{n}\frac{-a}{1-{\left[\frac{\vartheta \bar{G}(x_{i};\lambda_{k})}{1-\bar{\vartheta }\bar{G}(x_{i};\lambda_{k})}\right]}^{a}}{\left[\frac{\vartheta \bar{G}(x_{i};\lambda_{k})}{1-\bar{\vartheta }\bar{G}(x_{i};\lambda_{k})}\right]}^{a-1}\frac{\bar{G}(x_{i};\lambda_{k})[1-\bar{G}(x_{i};\lambda_{k})]}{{\left[1-\bar{\vartheta }\bar{G}(x_{i};\lambda_{k})\right]}^{2}}-(a+1)\sum_{j=1}^{m}\frac{\bar{G}(y_{j};\lambda_{k})}{1-\bar{\vartheta }\bar{G}(y_{j};\lambda_{k})}+(b_{2}-1)\sum_{j=1}^{m}\frac{-a}{1-{\left[\frac{\vartheta \bar{G}(y_{j};\lambda_{k})}{1-\bar{\vartheta }\bar{G}(y_{j};\lambda_{k})}\right]}^{a}}{\left[\frac{\vartheta \bar{G}(y_{j};\lambda_{k})}{1-\bar{\vartheta }\bar{G}(y_{j};\lambda_{k})}\right]}^{a-1}\frac{\bar{G}(y_{j};\lambda_{k})[1-\bar{G}(y_{j};\lambda_{k})]}{{\left[1-\bar{\vartheta }\bar{G}(y_{j};\lambda_{k})\right]}^{2}}$$ (5)

$$\frac{\partial \log L}{\partial a}=\frac{\left(n+m\right)}{a}+(n+m)\log\left(\vartheta \right)+\sum_{i=1}^{n}\log\left[\bar{G}(x_{i};\lambda_{k})\right]-\sum_{i=1}^{n}\log\left[1-\bar{\vartheta }\bar{G}(x_{i};\lambda_{k})\right]+(b_{1}-1)\sum_{i=1}^{n}\frac{1}{1-{\left[\frac{\vartheta \bar{G}(x_{i};\lambda_{k})}{1-\bar{\vartheta }\bar{G}(x_{i};\lambda_{k})}\right]}^{a}}{\left[\frac{\vartheta \bar{G}(x_{i};\lambda_{k})}{1-\bar{\vartheta }\bar{G}(x_{i};\lambda_{k})}\right]}^{a}\log\left[\frac{\vartheta \bar{G}(x_{i};\lambda_{k})}{1-\bar{\vartheta }\bar{G}(x_{i};\lambda_{k})}\right]+\sum_{j=1}^{m}\log\left[\bar{G}(y_{j};\lambda_{k})\right]-\sum_{j=1}^{m}\log\left[1-\bar{\vartheta }\bar{G}(y_{j};\lambda_{k})\right]+(b_{2}-1)\sum_{j=1}^{m}\frac{1}{1-{\left[\frac{\vartheta \bar{G}(y_{j};\lambda_{k})}{1-\bar{\vartheta }\bar{G}(y_{j};\lambda_{k})}\right]}^{a}}{\left[\frac{\vartheta \bar{G}(y_{j};\lambda_{k})}{1-\bar{\vartheta }\bar{G}(y_{j};\lambda_{k})}\right]}^{a}\log\left[\frac{\vartheta \bar{G}(y_{j};\lambda_{k})}{1-\bar{\vartheta }\bar{G}(y_{j};\lambda_{k})}\right]$$ (6)

$$\frac{\partial \log L}{\partial \lambda_{k}}=\sum_{i=1}^{n}\frac{1}{g(x_{i};\lambda_{k})}\frac{\partial g(x_{i};\lambda_{k})}{\partial \lambda_{k}}+(a-1)\sum_{i=1}^{n}\frac{1}{\bar{G}(x_{i};\lambda_{k})}\frac{\partial \bar{G}(x_{i};\lambda_{k})}{\partial \lambda_{k}}+(a+1)\sum_{i=1}^{n}\frac{\bar{\vartheta }}{1-\bar{\vartheta }\bar{G}(x_{i};\lambda_{k})}\frac{\partial \bar{G}(x_{i};\lambda_{k})}{\partial \lambda_{k}}+(b_{1}-1)\sum_{i=1}^{n}\frac{-a}{1-{\left[\frac{\vartheta \bar{G}(x_{i};\lambda_{k})}{1-\bar{\vartheta }\bar{G}(x_{i};\lambda_{k})}\right]}^{a}}{\left[\frac{\vartheta \bar{G}(x_{i};\lambda_{k})}{1-\bar{\vartheta }\bar{G}(x_{i};\lambda_{k})}\right]}^{a-1}\frac{\vartheta \frac{\partial \bar{G}(x_{i};\lambda_{k})}{\partial \lambda_{k}}[1-\bar{\vartheta }\bar{G}(x_{i};\lambda_{k})]+\bar{\vartheta }\frac{\partial \bar{G}(x_{i};\lambda_{k})}{\partial \lambda_{k}}\vartheta \bar{G}(x_{i};\lambda_{k})}{{\left[1-\bar{\vartheta }\bar{G}(x_{i};\lambda_{k})\right]}^{2}}+\sum_{j=1}^{m}\frac{1}{g(y_{j};\lambda_{k})}\frac{\partial g(y_{j};\lambda_{k})}{\partial \lambda_{k}}+(a-1)\sum_{j=1}^{m}\frac{1}{\bar{G}(y_{j};\lambda_{k})}\frac{\partial \bar{G}(y_{j};\lambda_{k})}{\partial \lambda_{k}}+(a+1)\sum_{j=1}^{m}\frac{\bar{\vartheta }}{1-\bar{\vartheta }\bar{G}(y_{j};\lambda_{k})}\frac{\partial \bar{G}(y_{j};\lambda_{k})}{\partial \lambda_{k}}+(b_{1}-1)\sum_{j=1}^{m}\frac{-a}{1-{\left[\frac{\vartheta \bar{G}(y_{j};\lambda_{k})}{1-\bar{\vartheta }\bar{G}(y_{j};\lambda_{k})}\right]}^{a}}{\left[\frac{\vartheta \bar{G}(y_{j};\lambda_{k})}{1-\bar{\vartheta }\bar{G}(y_{j};\lambda_{k})}\right]}^{a-1}\frac{\vartheta \frac{\partial \bar{G}(y_{j};\lambda_{k})}{\partial \lambda_{k}}[1-\bar{\vartheta }\bar{G}(y_{j};\lambda_{k})]+\bar{\vartheta }\frac{\partial \bar{G}(y_{j};\lambda_{k})}{\partial \lambda_{k}}\vartheta \bar{G}(y_{j};\lambda_{k})}{{\left[1-\bar{\vartheta }\bar{G}(y_{j};\lambda_{k})\right]}^{2}},k=1,\ldots ,l$$ (7)

Equating the partial derivatives to zero and then solving the equations numerically yields the maximum likelihood estimators for the parameters (*b*_1_, *b*_2_, *ϑ*, *a*, *λ*_*k*_).


$$\frac{\partial logL}{\partial b_{1}}=0,\frac{\partial logL}{\partial b_{2}}=0,\frac{\partial logL}{\partial \vartheta }=0,\frac{\partial logL}{\partial a}=0,\frac{\partial logL}{\partial \lambda_{k}}=0,$$

The maximum likelihood estimators for the parameters *b*_1_ *and b*_2_ are obtained as

$${\hat{b}}_{1}=\frac{-n}{\sum_{i=1}^{n}\log\left\{1-{\left[\frac{\vartheta \bar{G}(x_{i};\lambda_{k})}{1-\bar{\vartheta }\bar{G}(x_{i};\lambda_{k})}\right]}^{a}\right\}}$$ (8)

$${\hat{b}}_{2}=\frac{-m}{\sum_{j=1}^{m}\log\left\{1-{\left[\frac{\vartheta \bar{G}(y_{j};\lambda_{k})}{1-\bar{\vartheta }\bar{G}(y_{j};\lambda_{k})}\right]}^{a}\right\}}$$ (9)

The maximum likelihood estimates of the stress-strength reliability is obtained by substituting in Eq (1).


$$\hat{R}=\frac{{\hat{b}}_{1}}{{\hat{b}}_{1}+{\hat{b}}_{2}}$$ (10)

The partial derivatives with respect to the unknown parameters cannot be solved directly, so a simulation depends on methods like the Newton-Raphson method will be used to obtain the estimates of the unknown parameters and hence calculate the estimate of the stress-strength reliability.

## 5. Asymptotic Confidence Interval (A.C.I)

The observed Fisher information matrix of the stress strength reliability parameters is given by

$$I(\varphi )=I_{ij}=\left[-\frac{\partial^{2}\log L}{\partial \varphi_{i}\partial \varphi_{j}}\right],i,j=1,\ldots ,4+k,k=1,\ldots l$$

The elements of this matrix are obtained by differentiating the Eqs from (3) to (7) with respect to (*b*_1_, *b*_2_, *ϑ*, *a*, *λ*_*k*_), respectively, the results are obtained as

$$I_{11}=-\frac{\partial^{2}\log L}{\partial b_{1}^{2}},I_{22}=-\frac{\partial^{2}\log L}{\partial b_{2}^{2}},I_{33}=-\frac{\partial^{2}\log L}{\partial \vartheta^{2}},I_{44}=-\frac{\partial^{2}\log L}{\partial a^{2}},I_{55}=-\frac{\partial^{2}\log L}{\partial \lambda_{k}^{2}}$$

$$I_{12}=I_{21}=-\frac{\partial^{2}\log L}{\partial b_{1}\partial b_{2}},I_{13}=I_{31}=-\frac{\partial^{2}\log L}{\partial b_{1}\partial \vartheta },I_{14}=I_{41}=-\frac{\partial^{2}\log L}{\partial b_{1}\partial a},I_{15}=I_{51}=-\frac{\partial^{2}\log L}{\partial b_{1}\partial \lambda_{k}}$$

$$I_{23}=I_{32}=-\frac{\partial^{2}\log L}{\partial b_{2}\partial \vartheta },I_{24}=I_{42}=-\frac{\partial^{2}\log L}{\partial b_{2}\partial a},I_{25}=I_{52}=-\frac{\partial^{2}\log L}{\partial b_{2}\partial \lambda_{k}},I_{34}=I_{43}=-\frac{\partial^{2}\log L}{\partial \vartheta \partial a},$$

$$I_{35}=I_{53}=-\frac{\partial^{2}\log L}{\partial \vartheta \partial \lambda_{k}},I_{45}=I_{54}=-\frac{\partial^{2}\log L}{\partial a\partial \lambda_{k}},\ldots .,k=1,\ldots l$$

The asymptotic variances of the parameters (*b*_1_, *b*_2_, *ϑ*, *a*, *λ*_*k*_) are given by

$$Var(b_{1})=I_{11}^{-1},Var(b_{2})=I_{22}^{-1},Var(\vartheta )=I_{33}^{-1},Var(a)=I_{44}^{-1},Var(\lambda_{k})=I_{55}^{-1},\ldots ,k=1,\ldots l$$

The asymptotic variance of an estimate $\hat{R}$ is given by

$$Var(\hat{R})=I_{11}^{-1}{\left(\frac{\partial \hat{R}}{\partial {\hat{b}}_{1}}\right)}^{2}+I_{22}^{-1}{\left(\frac{\partial \hat{R}}{\partial {\hat{b}}_{2}}\right)}^{2}+2I_{12}^{-1}\left(\frac{\partial \hat{R}}{\partial {\hat{b}}_{1}}\right)\left(\frac{\partial \hat{R}}{\partial {\hat{b}}_{2}}\right)=\frac{{\hat{b}}_{1}^{2}{\hat{b}}_{2}^{2}}{n{\left({\hat{b}}_{1}+{\hat{b}}_{2}\right)}^{4}}\left(\frac{1}{n}+\frac{1}{m}\right)$$

$As\;n\to \infty ,m\to \infty ,\frac{\hat{R}-R}{Var(\hat{R})}\to N(0,1)$ and the asymptotic 100(1−*δ*)% confidence interval of R is given by

$$\hat{R}\pm z_{1-\frac{\delta }{2}}\sqrt{Var(\hat{R})}=\hat{R}\pm z_{1-\frac{\delta }{2}}\frac{{\hat{b}}_{1}{\hat{b}}_{2}}{{\left({\hat{b}}_{1}+{\hat{b}}_{2}\right)}^{2}}\sqrt{\frac{1}{n}+\frac{1}{m}}$$

The asymptotic confidence interval is used for large samples and do not perform well for small samples. So, the bootstrap confidence interval method will be proposed.

## 6. Bootstrap Confidence Interval (B.C.I.)

The bootstrap method for constructing confidence intervals is illustrated by the following algorithm.

**Step 1:** Generate independent random samples *x*_1_, *x*_2_,…,*x*_*n*_ from EGMO-G(*b*_1_, *ϑ*, *a*, *λ*_*k*_) and *y*_1_, *y*_2_,…,*y*_*m*_ from EGMO-G(*b*_2_, *ϑ*, *a*, *λ*_*k*_)

**Step 2:** Generate new samples $x_{1}^{*},x_{2}^{*},\ldots ,x_{n}^{*}$ and $y_{1}^{*},y_{2}^{*},\ldots ,y_{m}^{*}$ taken by sampling with replacement from the samples in step (1) and compute the MLE estimate of stress strength reliability ${\hat{R}}^{*}$ by using Eq (9).

**Step 3:** Repeat step 2, S times to obtain the estimates $\left\{{\hat{R}}_{1}^{*},{\hat{R}}_{2}^{*},\ldots ,{\hat{R}}_{B}^{*}\right\}$.

**Step 4:** Rearrange the estimates obtained in step (3) such that ${\hat{R}}_{1}^{*}<{\hat{R}}_{2}^{*}<\cdots <{\hat{R}}_{S}^{*}$. The 100(1−*δ*)% bootstrap confidence interval is given by

$$\left[{\hat{R}}_{S(\delta /2)}^{*},{\hat{R}}_{S(1-\delta /2)}^{*}\right]$$

## 7. Bayesian estimation

Bayesian estimation method will be discussed into cases. The first case when the parameters are unknown and the second case when the parameters are known.

### Case I: When the parameters are unknown

The Bayesian estimator for the stress-strength reliability will be obtained assuming that the parameters *b*_1_, *b*_2_, *ϑ*, *a and λ*_*k*_ are independent random variables with prior follow gamma distribution as follows

$$b_{1}∼Gamma(\xi_{1},\eta_{1})$$

$$b_{2}∼Gamma(\xi_{2},\eta_{2})$$

$$\vartheta ∼Gamma(\xi_{3},\eta_{3})$$

$$a∼Gamma(\xi_{4},\eta_{4})$$

$$\lambda_{k}∼Gamma(\xi_{5k},\eta_{5k}),k=1,2,\ldots ,l$$

The joint posterior density function of *b*_1_, *b*_2_, *ϑ*, *a and λ*_*k*_ given the data (x, y) is given by

$$\Pi (b_{1},b_{2},\vartheta ,a,\lambda_{k}|x,y)\propto b_{1}^{n+\eta_{1}-1}e^{-\xi_{1}b_{1}}b_{2}^{m+\eta_{2}-1}e^{-\xi_{2}b_{2}}\vartheta^{a(n+m)+\eta_{3}-1}e^{-\xi_{3}\vartheta }a^{n+m+\eta_{4}-1}e^{-\xi_{4}a}\prod_{k=1}^{l}\lambda_{k}^{\eta_{k}-1}e^{-\xi_{5k}\lambda_{k}}$$

$$\prod_{i=1}^{n}\frac{ab_{1}\vartheta^{a}g(x_{i};\lambda_{k}){\left[\bar{G}(x_{i};\lambda_{k})\right]}^{a-1}}{{\left[1-\bar{\vartheta }\bar{G}(x_{i};\lambda_{k})\right]}^{a+1}}{\left\{1-{\left[\frac{\vartheta \bar{G}(x_{i};\lambda_{k})}{1-\bar{\vartheta }\bar{G}(x_{i};\lambda_{k})}\right]}^{a}\right\}}^{b_{1}-1}$$

$$\prod_{j=1}^{m}\frac{ab_{2}\vartheta^{a}g(y_{j};\lambda_{k}){\left[\bar{G}(y_{j};\lambda_{k})\right]}^{a-1}}{{\left[1-\bar{\vartheta }\bar{G}(y_{j};\lambda_{k})\right]}^{a+1}}{\left\{1-{\left[\frac{\vartheta \bar{G}(y_{j};\lambda_{k})}{1-\bar{\vartheta }\bar{G}(y_{j};\lambda_{k})}\right]}^{a}\right\}}^{b_{2}-1}$$

$$\Pi (b_{1},b_{2},\vartheta ,a,\lambda_{k}|x,y)\propto b_{1}^{n+\eta_{1}-1}e^{-b_{1}H_{1}}b_{2}^{m+\eta_{2}-1}e^{-b_{2}H_{2}}\vartheta^{a(n+m)+\eta_{3}-1}e^{-\xi_{3}\vartheta }a^{n+m+\eta_{4}-1}e^{-aH_{3}}\prod_{k=1}^{l}\lambda_{k}^{\eta_{5k}-1}e^{-\xi_{5k}\lambda_{k}+H_{4}}$$

where

$$H_{1}=\xi_{1}-\sum_{i=1}^{n}\log\left\{1-{\left[\frac{\vartheta \bar{G}(x_{i};\lambda_{k})}{1-\bar{\vartheta }\bar{G}(x_{i};\lambda_{k})}\right]}^{a}\right\}$$

$$H_{2}=\xi_{2}-\sum_{j=1}^{m}\log\left\{1-{\left[\frac{\vartheta \bar{G}(y_{j};\lambda_{k})}{1-\bar{\vartheta }\bar{G}(y_{j};\lambda_{k})}\right]}^{a}\right\}$$

$$H_{3}=\xi_{4}-\sum_{i=1}^{n}\log\left[\bar{G}(x_{i};\lambda_{k})\right]-\sum_{j=1}^{m}\log\left[\bar{G}(y_{j};\lambda_{k})\right]+\sum_{i=1}^{n}\log\left[1-\bar{\vartheta }\bar{G}(x_{i};\lambda_{k})\right]+\sum_{j=1}^{m}\log\left[1-\bar{\vartheta }\bar{G}(y_{j};\lambda_{k})\right]$$

$$H_{4}=\sum_{i=1}^{n}\log\left[g(x_{i};\lambda_{k})\right]-\sum_{i=1}^{n}\log\left[\bar{G}(x_{i};\lambda_{k})\right]-\sum_{i=1}^{n}\log\left[1-\bar{\vartheta }\bar{G}(x_{i};\lambda_{k})\right]-\sum_{i=1}^{n}\log\left\{1-{\left[\frac{\vartheta \bar{G}(x_{i};\lambda_{k})}{1-\bar{\vartheta }\bar{G}(x_{i};\lambda_{k})}\right]}^{a}\right\}+\sum_{j=1}^{m}\log\left[g(y_{j};\lambda_{k})\right]-\sum_{j=1}^{m}\log\left[\bar{G}(y_{j};\lambda_{k})\right]-\sum_{j=1}^{m}\log\left[1-\bar{\vartheta }\bar{G}(y_{j};\lambda_{k})\right]-\sum_{j=1}^{m}\log\left\{1-{\left[\frac{\vartheta \bar{G}(y_{j};\lambda_{k})}{1-\bar{\vartheta }\bar{G}(y_{j};\lambda_{k})}\right]}^{a}\right\}$$

The marginal posterior distributions of *b*_1_, *b*_2_, *ϑ*, *a and λ*_*k*_ can be deduced as

$$\Pi_{1}^{*}(b_{1}|\vartheta ,\lambda_{k},x)\propto Gamma(n+\eta_{1},H_{1})$$

$$\Pi_{2}^{*}(b_{2}|\vartheta ,\lambda_{k},y)\propto Gamma(m+\eta_{2},H_{2})$$

$$\Pi_{3}^{*}(\vartheta |a,\lambda_{k},x,y)\propto Gamma(a(n+m)+\eta_{3},\xi_{3})$$

$$\Pi_{4}^{*}(a|\lambda_{k},x,y)\propto Gamma(n+m+\eta_{4},H_{3})$$

$$\Pi_{5}^{*}(\lambda_{k}|x,y)\propto \lambda_{k}^{\eta_{5k}-1}e^{-\xi_{5k}\lambda_{k}+H_{4}},k=1,2,\ldots ,l$$

It is obvious that seen that posterior samples for *b*_1_, *b*_2_, *ϑ and a* can be generated using gamma distribution. However, *λ*_*k*_ cannot be directly simulated from its posterior distribution as it is not in known form and in this case the Metropolis-Hastings algorithm can be applied to simulate random samples from the posterior density of *λ*_*k*_.

The Bayesian estimator of the reliability function R under the squared error loss function using the posterior mean is given by

$$R_{SEL}=E(R|x,y)=\int_{0}^{\infty }\int_{0}^{\infty }R\Pi (b_{1},b_{2},\vartheta ,a,\lambda_{k}|x,y)db_{1}db_{2}d\vartheta dad\lambda_{k}$$

This integral has no analytical solution and the Markov Chain Monte Carlo (MCMC) simulation method can be applied to obtain the Bayesian estimation of the stress-strength reliability. In the method of the Markov chain Monte Carlo, samples are generated from the posterior density function and in turn to compute the Bayes estimates of the reliability function.

### 7.1 MCMC method

Markov chain Monte Carlo (MCMC) method comprises a class of algorithms for sampling from a probability distribution. By constructing a Markov chain that has the desired distribution as its equilibrium distribution, one can obtain a sample of the desired distribution by recording states from the chain. The more steps that are included, the more closely the distribution of the sample matches the actual desired distribution. Various algorithms exist for constructing chains, including the Metropolis–Hastings algorithm. The steps of applying MCMC method and the Metropolis–Hastings algorithm are presented as follows.

**Step1:** Choose initial values $b_{1}^{0},b_{2}^{0},\vartheta^{0},a^{0},\lambda_{k}^{0}$

**Step 2:** Set *t* =1

**Step 3:** Generate $b_{1}^{\left(t\right)}$ from *Gamma*(*n*+*η*_1_, *H*_1_)

**Step 4:** Generate $b_{2}^{\left(t\right)}$ from *Gamma*(*m*+*η*_2_, *H*_2_)

**Step 5:** Generate *ϑ*^(*t*)^ from *Gamma*(*a*(*n*+*m*)+*η*_3_, ξ_3_)

**Step 6:** Generate *a*^(*t*)^ from *Gamma*(*n*+*m*+*η*_4_,*H*_3_)

**Step 7:** Generate $\lambda_{k}^{\left(t\right)}$ from $\Pi_{5}^{*}(\lambda_{k}|x,y)$ using MH algorithm as the following

- Generate proposals $\lambda_{k}^{*}$ from $N\left(\lambda_{k}^{\left(t-1\right)},Var(\lambda_{k}^{\left(t-1\right)})\right),l=1,\ldots ,k$.
- Evaluate the acceptance probabilities
$$z_{k}=min\left\{1,\frac{\Pi_{5}^{*}(\lambda_{k}^{*}|x,y)}{\Pi_{5}^{*}(\lambda_{k}^{\left(t-1\right)}|x,y)}\right\},k=1,\ldots ,l$$
- Generate *u*_*k*_ from *Uniform*(0, 1).
- If *u*_*k*_<*z*_*k*_, accept the proposal and set $\lambda_{k}^{\left(t\right)}=\lambda_{k}^{*}$, else set $\lambda_{k}^{\left(t\right)}=\lambda_{k}^{\left(t-1\right)}$

**Step 8:** Compute *R*^(*t*)^ from Eq (9)

**Step 9:** Set *t* = *t*+1

**Step 10:** Repeat steps from 3 to 9, T times.

**Step 11:** Stop for sufficiently large value of T, the Bayes estimate of the stress-strength reliability function under the squared error loss will be given as

$${\hat{R}}_{B}=\frac{1}{T}\sum_{t=1}^{T}R^{\left(t\right)}$$

**Step 12:** To construct the credible interval for R, order *R*^(*t*)^ as *R*^(1)^<*R*^(2)^<⋯<*R*^(*T*)^. Then a 100(1−*ε*)% credible interval of R becomes $\left[{\hat{R}}_{B,T(\varepsilon /2)},{\hat{R}}_{B,T(1-\varepsilon /2)}^{*}\right]$

#### Case II: When the parameters are known

In this case the Bayes estimator of the stress-strength reliability under the squared error loss will be given by

$${\hat{R}}_{B}=\int_{0}^{\infty }\int_{0}^{\infty }R\Pi (b_{1},b_{2}|x,y)db_{1}db_{2}$$

$${\hat{R}}_{B}=\frac{H_{1}^{n+\eta_{1}}H_{2}^{m+\eta_{2}}}{\Gamma (n+\eta_{1})\Gamma (m+\eta_{2})}\int_{0}^{\infty }\int_{0}^{\infty }\frac{b_{1}}{{b_{1}+b}_{2}}b_{1}^{n+\eta_{1}-1}b_{2}^{m+\eta_{2}-1}e^{-H_{1}b_{1}}e^{-H_{2}b_{2}}db_{1}db_{2}$$

Let

$$w_{1}=\frac{b_{1}}{{b_{1}+b}_{2}}\;and\;w_{2}={b_{1}+b}_{2}$$

where 0<*w*_1_<1, *w*_2_>0. Hence, the Bayes estimator of the stress-strength reliability will be given by

$${\hat{R}}_{B}=\frac{{\left(1-\psi \right)}^{n+\eta_{1}}}{\beta (n+\eta_{1},m+\eta_{2})}\int_{0}^{1}w_{1}^{n+\eta_{1}}{\left(1-w_{1}\right)}^{m+\eta_{2}-1}{\left(1-\psi w_{1}\right)}^{-c}dw_{1}$$

Where

$$c=n+\eta_{1}+m+\eta_{2}\;and\;\psi =1-\left(\frac{w_{1}}{w_{2}}\right)$$

The Bayes estimator of the stress strength reliability is deduced as

$${\hat{R}}_{B}=\left\{ \begin{array}{c} \frac{{\left(1-\psi \right)}^{n+\eta_{1}}(n+\eta_{1})}{c}{2F}_{1}(c,n+\eta_{1}+1,c+1,\psi ),\;if\;|\psi |<1\\ \frac{n+\eta_{1}}{c{\left(1-\psi \right)}^{m+\eta_{2}}}{2F}_{1}(c,m+\eta_{2},c+1,\frac{\psi }{\psi -1}),\;if\;\psi <-1 \end{array}\right.$$

In some situations, it is difficult to find the stress strength reliability from the previous relations and in this case, the MCMC method can be used to find an estimate for the stress strength reliability based on simulated random samples and the steps are given as follows.

**Step1:** Choose initial values $b_{1}^{0},b_{2}^{0},a^{0},\vartheta^{0},\lambda_{k}^{0}$

**Step 2:** Set *t* =1

**Step 3:** Generate *b*_1_ from *Gamma*(*n*+*η*_1_, *H*_1_)

**Step 4:** Generate *b*_2_ from *Gamma*(*m*+*η*_2_, *H*_2_)

**Step 5:** Compute *R*^*t*^ from Eq (9)

**Step 6:** Set *t* = *t*+1

**Step 7:** Repeat steps from 3 to 6, T times,

**Step 8:** Stop for sufficiently large value of T, the Bayes estimate of the stress-strength reliability function under the squared error loss will be given as

$${\hat{R}}_{B}=\frac{1}{T}\sum_{t=1}^{T}R^{\left(t\right)}$$

### 7.2 Credible interval

The credible interval for the stress strength reliability can be deduced as follows. From the relations between the gamma distribution and chi-square distribution, it can be shown that

$$2H_{1}b_{1}=2\left(\xi_{1}-\sum_{i=1}^{n}\log\left\{1-{\left[\frac{\vartheta \bar{G}(x_{i};\lambda_{k})}{1-\bar{\vartheta }\bar{G}(x_{i};\lambda_{k})}\right]}^{a}\right\}\right)b_{1}∼\chi_{2(n+\eta_{1})}^{2}$$

and

$$2H_{2}b_{2}=2\left(\xi_{2}-\sum_{j=1}^{m}\log\left\{1-{\left[\frac{\vartheta \bar{G}(y_{j};\lambda_{k})}{1-\bar{\vartheta }\bar{G}(y_{j};\lambda_{k})}\right]}^{a}\right\}\right)b_{2}∼\chi_{2(m+\eta_{2})}^{2}$$

The posterior distribution of R can be written as

$${\left[1+\frac{(m+\eta_{2})H_{1}}{(n+\eta_{1})H_{2}}F(2(m+\eta_{2}),2(n+\eta_{1}))\right]}^{-1}$$

And therefore a 100(1−*ε*)% credible interval for R will be given by

$$\left({\left[1+\frac{(m+\eta_{2})H_{1}}{(n+\eta_{1})H_{2}}F_{\frac{\varepsilon }{2},2(m+\eta_{2}),2(n+\eta_{1})}\right]}^{-1},{\left[1+\frac{(m+\eta_{2})H_{1}}{(n+\eta_{1})H_{2}}F_{1-\frac{\varepsilon }{2},2(m+\eta_{2}),2(n+\eta_{1})}\right]}^{-1}\right)$$

## 8. Simulation study

Monte Carlo simulation is performed with replications of 1000 samples. The simulated samples are generated using different values of the parameters from the exponentiated generalized Marshall Olkin Weibull distribution (EGMO-W). The simulated samples are taken of different sizes: (5, 5), (10, 10), (20, 20), (30, 30), (50, 50) and (100, 100). The results for the maximum likelihood estimates of the strength-stress reliability with the mean squared error values, the 95% bootstrap confidence intervals with (B = 1000) and the 95% asymptotic confidence intervals are obtained. The MCMC method is performed with T = 1000, the Bayes estimates of the strength-stress reliability and the 95% credible intervals are deduced in two cases: The first case of prior-I (ξ_i_ = 2, *η*_*i*_ = 3, *i* = 1,…5) and the second case of prior-II (ξ_i_ = 1, *η*_*i*_ = 10, *i* = 1,…5). All the simulations and computations were performed using the software program R. The results are presented in Tables 1–4. Steps of applying the Monte Carlo simulation is illustrated as follows.

**Step 1**: Set initial values of the parameters (*b*_1_, *b*_2_, *ϑ*, *a*, *γ*, *β*).

**Step 2**: Choose the samples sizes (n, m).

**Step 3**: Generate random values of the random variables *X*_*i*_ and *Y*_*j*_ at the initial values of the parameters (*b*_1_, *b*_2_, *ϑ*, *a*, *γ*, *β*) by applying the following inversion formula method

Xi=G−1{ϑ[1−(1−ui1b1)1a]ϑ+ϑ¯[1−(1−ui1b1)1a]},0<ui<1,i=1,2,…,n

Yj=G−1{ϑ[1−(1−sj1b2)1a]ϑ+ϑ¯[1−(1−sj1b2)1a]},0<sj<1,j=1,2,…,m

For EGMO-W distribution the inversion formula will be given by

Xi=[−βγlog{ϑ[1−(1−ui1b1)1a]ϑ+ϑ¯[1−(1−ui1b1)1a]}]1γ,0<ui<1,i=1,2,…,n

Yj=[−βγlog{ϑ[1−(1−sj1b2)1a]ϑ+ϑ¯[1−(1−sj1b2)1a]}]1γ,0<sj<1,j=1,2,…,m

**Step 4**: Solve the differential Eqs (3)–(5) and using Eqs (8) and (9) to obtain the estimates of the parameters $\left({\hat{b}}_{1},{\hat{b}}_{2},\hat{\vartheta },\hat{a},\hat{\gamma },\hat{\beta }\right)$ by using the Newton-Raphson method and the aid of software program R.

**Step 5**: Obtain the estimate of the stress-strength reliability by substituting in Eq (10).

**Step 6**: Repeat steps from 3 to 5, 1000 times. In each time of simulation, the same values of the initial parameters and the same samples sizes are considered but the values of generating random samples are varying each time. So, we have 1000 values of estimates of the stress-strength reliability $\hat{R}$. The mean squared error (MSE) can be obtained from the following relation

$$MSE=\frac{\sum_{i=1}^{1000}{\left({\hat{R}}_{i}-R\right)}^{2}}{1000}$$

**Table 1:** Simulation results for MLE and Bayesian estimation with MSE when *a* = *b*_1_ = *b*_2_ = *ϑ* = *γ* = *β* = 0.5.

n	m	R (true)	Case	MLE estimation		Bayesian estimation			
				$\hat{R}$	MSE	Prior I		Prior II	
						${\hat{R}}_{B}$	MSE	${\hat{R}}_{B}$	MSE
5	5	0.5000	(I)	0.448618	0.076185	0.504086	0.018399	0.504226	0.010587
10	10			0.376515	0.076014	0.497621	0.009842	0.499643	0.008072
20	20			0.343232	0.073981	0.495885	0.004346	0.498929	0.003951
30	30			0.307502	0.073907	0.496269	0.003126	0.497799	0.002726
50	50			0.220569	0.070867	0.497380	0.001887	0.497666	0.001658
100	100			0.087001	0.064241	0.497971	0.001079	0.499830	0.000800
5	5	0.5000	(II)	0.314211	0.057954	0.502845	0.026632	0.501145	0.011346
10	10			0.214301	0.057370	0.501770	0.019505	0.501036	0.010426
20	20			0.285712	0.057179	0.500264	0.014573	0.499613	0.007915
30	30			0.283348	0.057122	0.499867	0.011425	0.499266	0.006788
50	50			0.257732	0.057063	0.499615	0.007669	0.498784	0.005424
100	100			0.249249	0.056940	0.494586	0.003978	0.497248	0.003447

**Table 2:** Results for confidence, bootstrap confidence and credible intervals when *a* = *b*_1_ = *b*_2_ = *ϑ* = *γ* = *β* = 0.5.

n	m	Case	Confidence interval				Credible interval			
			A.C.I.	Length	B.C.I.	Length	Prior I		Prior II	
							${\hat{R}}_{B}$	Length	${\hat{R}}_{B}$	Length
5	5	(I)	[0.141987, 0.755248]	0.613261	[0.010057, 0.923073]	0.913016	[0.228731, 0.788758]	0.560027	[0.301099, 0.713104]	0.412004
10	10		[0.170746, 0.582284]	0.411538	[0.014241, 0.917009]	0.902768	[0.302925, 0.694075]	0.391149	[0.318083, 0.672477]	0.354394
20	20		[0.203513, 0.482951]	0.279438	[0.019221, 0.830878]	0.811657	[0.367382, 0.630274]	0.262891	[0.378536, 0.626606]	0.248069
30	30		[0.199738, 0.415267]	0.215529	[0.022777, 0.788644]	0.765867	[0.383185, 0.605105]	0.221919	[0.397012, 0.601881]	0.204868
50	50		[0.153177, 0.287961]	0.134784	[0.025494, 0.757860]	0.732366	[0.412466, 0.585917]	0.173450	[0.416195, 0.576022]	0.159827
100	100		[0.064984, 0.109019]	0.044035	[0.031745, 0.625062]	0.593316	[0.440859, 0.557361]	0.116501	[0.444337, 0.553112]	0.108774
5	5	(II)	[0.04709, 0.581326]	0.534230	[0.185412, 0.486756]	0.301344	[0.18598, 0.818649]	0.632662	[0.291696, 0.703403]	0.411707
10	10		[0.06671, 0.361889]	0.295176	[0.14227, 0.377379]	0.235108	[0.23860, 0.771346]	0.532745	[0.301834, 0.699049]	0.397214
20	20		[0.15922, 0.412202]	0.252981	[0.14998, 0.295455]	0.145471	[0.260377, 0.717884]	0.457507	[0.329820, 0.682645]	0.352824
30	30		[0.18058, 0.386111]	0.205527	[0.21911, 0.338584]	0.119469	[0.294511, 0.713936]	0.419425	[0.337819, 0.659044]	0.321225
50	50		[0.18274, 0.332724]	0.149984	[0.23737, 0.325332]	0.087958	[0.33063, 0.675477]	0.344840	[0.35670, 0.633283]	0.276582
100	100		[0.19738, 0.301117]	0.103736	[0.21773, 0.289639]	0.071905	[0.374662, 0.626659]	0.251997	[0.38206, 0.612575]	0.230509

**Table 3:** Simulation results for MLE and Bayesian estimation with MSE when *a* = 0.5, *b*_1_ = 1.5, *b*_2_ = 2.5, *ϑ* = 0.5, *γ* = 3.5, *β* = 1.5.

n	m	R (true)	Case	MLE estimation		Bayesian estimation			
				$\hat{R}$	MSE	Prior I		Prior II	
						${\hat{R}}_{B}$	MSE	${\hat{R}}_{B}$	MSE
5	5	0.3750	(I)	0.357875	0.027337	0.474308	0.013360	0.471956	0.013172
10	10			0.397694	0.025373	0.473102	0.011041	0.471857	0.011184
20	20			0.318187	0.022655	0.471577	0.009901	0.469100	0.009603
30	30			0.299166	0.018206	0.471627	0.009700	0.468618	0.009197
50	50			0.285734	0.015658	0.472403	0.009686	0.469427	0.009132
100	100			0.170185	0.009687	0.472701	0.009638	0.469281	0.008996
5	5	0.3750	(II)	0.361605	0.014743	0.499663	0.037874	0.499819	0.024971
10	10			0.304985	0.014367	0.490534	0.027250	0.494306	0.022938
20	20			0.285661	0.013656	0.487328	0.021535	0.488809	0.019498
30	30			0.224819	0.013623	0.480378	0.018384	0.486915	0.017510
50	50			0.266693	0.013522	0.478072	0.015297	0.481756	0.015074
100	100			0.277626	0.013495	0.475464	0.012557	0.480844	0.013326

**Table 4:** Results for confidence, bootstrap confidence and credible intervals when *a* = 0.5, *b*_1_ = 1.5, *b*_2_ = 2.5, *ϑ* = 0.5, *γ* = 3.5, *β* = 1.5.

n	m	Case	Confidence interval				Credible interval			
			A.C.I.	Length	B.C.I.	Length	Prior I		Prior II	
							${\hat{R}}_{B}$	Length	${\hat{R}}_{B}$	Length
5	5	(I)	[0.073011, 0.642738]	0.569727	[0.032473, 0.750153]	0.717679	[0.360648, 0.591913]	0.231264	[0.352277, 0.596854]	0.244576
10	10		[0.187734, 0.607654]	0.419920	[0.039622, 0.696295]	0.656672	[0.394217, 0.545093]	0.150876	[0.386400, 0.553404]	0.167004
20	20		[0.183723, 0.452650]	0.268926	[0.047815, 0.656072]	0.608256	[0.423375, 0.514825]	0.091450	[0.415981, 0.521636]	0.105654
30	30		[0.193060, 0.405271]	0.212211	[0.035506, 0.616601]	0.581094	[0.430411, 0.507758]	0.077347	[0.425857, 0.506946]	0.081088
50	50		[0.205731, 0.365737]	0.160006	[0.038861, 0.581814]	0.542953	[0.442243, 0.498661]	0.056418	[0.439446, 0.497196]	0.057750
100	100		[0.131040, 0.209330]	0.078289	[0.107003, 0.576527]	0.469524	[0.453269, 0.491371]	0.038102	[0.447892, 0.489689]	0.041796
5	5	(II)	[0.075444, 0.647766]	0.572321	[0.189580, 0.492777]	0.303196	[0.196914, 0.783690]	0.586775	[0.313680, 0.696105]	0.382425
10	10		[0.119185, 0.490784]	0.371598	[0.129619, 0.339988]	0.210368	[0.261860, 0.726424]	0.464564	[0.314024, 0.683372]	0.369348
20	20		[0.159184, 0.412138]	0.252954	[0.175367, 0.301129]	0.125762	[0.302485, 0.663840]	0.361355	[0.317927, 0.644770]	0.326842
30	30		[0.136623, 0.313014]	0.176391	[0.206354, 0.329974]	0.123620	[0.312101, 0.647605]	0.335504	[0.340430, 0.626938]	0.286508
50	50		[0.190030, 0.343355]	0.153325	[0.197900, 0.289288]	0.091388	[0.344624, 0.611626]	0.267001	[0.369679, 0.600175]	0.230495
100	100		[0.222037, 0.333216]	0.111179	[0.236600, 0.298231]	0.061630	[0.377831, 0.571235]	0.193403	[0.393370, 0.571494]	0.178124

In Table 1, simulation results are obtained for the stress strength reliability estimates by applying the maximum likelihood method and Bayesian estimation method in case of prior I and prior II when the values of the parameters are given by *a* = *b*_1_ = *b*_2_ = *ϑ* = *γ* = *β* = 0.5. In Table 2, results for confidence, bootstrap confidence and credible intervals in case of prior I and prior II are deduced when the values of the parameters are given by *a* = *b*_1_ = *b*_2_ = *ϑ* = *γ* = *β* = 0.5.

In Table 3, simulation results are obtained for the stress strength reliability estimates by applying the maximum likelihood method and Bayesian estimation method in case of prior I and prior II when the values of the parameters are given by *a* = 0.5, *b*_1_ = 1.5, *b*_2_ = 2.5, *ϑ* = 0.5, *γ* = 3.5, *β* = 1.5. In Table 4, results for confidence, bootstrap confidence and credible intervals in case of prior I and prior II are deduced when the values of the parameters are given by *a* = 0.5, *b*_1_ = 1.5, *b*_2_ = 2.5, *ϑ* = 0.5, *γ* = 3.5, *β* = 1.5. All results obtained in Tables 1–4 are performed in case of the parameters are unknown (case I) and in case of the parameters are known.

From the results obtained in Tables 1–4, it can be concluded the following:

1. The values of the mean squared errors decrease as the sample sizes increase.
2. The lengths of the asymptotic confidence intervals, bootstrap confidence intervals and credible intervals decrease as the sample sizes increase.
3. Stress strength reliability estimates obtained in case of Bayesian estimation method is greater than stress strength reliability estimates obtained in case of maximum likelihood method.

## 9. Real data Application 1

A real data for the confirmed, recovered cases and deaths for covid 19 in Saudi Arabia from 1 April 2020 to 15 May 2020 are given in Table 5. These data obtained from the website (https://datahub.io/core/ covid-19#resource-covid-19_zip/). It is considered that the stress and strength follow exponentiated generalized Marshall Olkin exponential distribution (EGMO-E) and death rate represents the stress and the recovery rate represents the strength, where

$$Death\;rate=\frac{Deaths}{Confirmed\;cases}$$

$$Recovery\;rate=\frac{Recovered\;cases}{Confirmed\;cases}$$

Calculations of recovery and death rates based on the confirmed, recovered cases and deaths of covid 19 data are presented in Table 5. Summary of the measures of the death and recovery rate is illustrated in Table 6.

**Table 5:** Recovery and death rates based on the confirmed, recovered cases and deaths of covid 19.

Confirmed cases	Recovered cases	Deaths	Recovery rate	Death rate
1720	264	16	0.1534	0.930×10^−2^
1885	328	21	0.1740	1.114×10^−2^
2039	351	25	0.1721	1.226×10^−2^
2179	420	29	0.1927	1.330×10^−2^
2402	488	34	0.2031	1.415×10^−2^
2605	551	38	0.2115	1.458×10^−2^
2795	615	41	0.2200	1.466×10^−2^
2932	631	41	0.2152	1.398×10^−2^
3287	666	44	0.2026	1.338×10^−2^
3651	685	47	0.1876	1.287×10^−2^
4033	720	52	0.1785	1.289×10^−2^
4462	761	59	0.1705	1.322×10^−2^
4934	805	65	0.1631	1.317×10^−2^
5369	889	73	0.1655	1.359×10^−2^
5862	931	79	0.1588	1.347×10^−2^
6380	990	83	0.1551	1.300×10^−2^
7142	1049	87	0.1468	1.218×10^−2^
8274	1329	92	0.1606	1.111×10^−2^
9362	1398	97	0.1493	1.036×10^−2^
10484	1490	103	0.1421	0.982×10^−2^
11631	1640	109	0.1410	0.937×10^−2^
12772	1812	114	0.1418	0.892×10^−2^
13930	1925	121	0.1381	0.868×10^−2^
15102	2049	127	0.1356	0.840×10^−2^
16299	2215	136	0.1358	0.834×10^−2^
17522	2357	139	0.1345	0.793×10^−2^
18811	2531	144	0.1345	0.765×10^−2^
20077	2784	152	0.1386	0.757×10^−2^
21402	2953	157	0.1379	0.733×10^−2^
22753	3163	162	0.1390	0.711×10^−2^
24097	3555	169	0.1475	0.701×10^−2^
25459	3765	176	0.1478	0.691×10^−2^
27011	4134	184	0.1530	0.681×10^−2^
28656	4476	191	0.1561	0.666×10^−2^
30251	5431	200	0.1795	0.661×10^−2^
31938	6783	209	0.2123	0.654×10^−2^
33731	7798	219	0.2311	0.649×10^−2^
35432	9120	229	0.2573	0.646×10^−2^
37136	10144	239	0.2731	0.643×10^−2^
39048	11457	246	0.2934	0.629×10^−2^
41014	12737	255	0.3105	0.621×10^−2^
42925	15257	264	0.3554	0.615×10^−2^
44830	17622	273	0.3930	0.608×10^−2^
46869	19051	283	0.4064	0.603×10^−2^
49176	21869	292	0.4447	0.593×10^−2^

**Table 6:** Summary of the measures of the death and recovery rate.

Measure	Recovery rate	Death rate
**Mean**	0.009563111	0.1968978
**Median**	0.00868	0.1655
**Standard Deviation**	0.003033138	0.07769018
**Standard Error**	0.0004521535	0.001726448
**Skewness**	0.3257723	1.694957
**Kurtosis**	-1.558225	2.054589

The goodness of fit for the exponentiated generalized Marshall Olkin exponential distribution (EGMO-E) is compared with the generalized Marshall Olkin exponential distribution (GMO-E), the exponentiated generalized exponential distribution (EG-E), the Marshall Olkin exponential distribution (MO-E) and the exponential distribution (E). The results of the goodness of fit for the death and recovery rate of covid 19 data are shown in Table 7.

**Table 7:** MLE estimates, Log L, AIC, BIC and KS for the death and recovery rate.

Data	Distribution	Parameters	Estimates	Standard error	Log L	AIC	KS	P-value
Death rate	EGMO-E	*a* *b*_1_ *ϑ* *λ*	3.553×10^−1^ 3.089 1.194×10^2^ 1.039×10^3^	5.487×10^−2^ 7.536×10^−1^ 6.016 3.213×10^−1^	200.514	-393.029	0.1567	0.1971
	GMO-E	*b*_1_ *ϑ* *λ*	4.008×10^−1^ 1.450×10^4^ 7.935×10^2^	5.981×10^−2^ 5.981×10^−1^ 5.981×10^−2^	191.953	-377.906	0.1848	0.0809
	EG-E	*a* *ϑ* *λ*	618.2039 7109.0995 242.8883	0.1551 0.1015 0.1311	193.494	-380.989	0.2443	0.0075
	MO-E	*ϑ* *λ*	134.924 524.524	5.932 2.422	195.361	-386.723	0.1524	0.2229
	E	*λ*	104.573	5.932	164.242	-326.485	0.4621	2.564×10^−9^
Recovery rate	EGMO-E	*a* *b*_2_ *ϑ* *λ*	0.3224 2.8792 474.5942 59.9997	0.0618 0.9162 8.8533 5.3767	67.036	-126.072	0.1450	0.3000
	GMO-E	*b*_2_ *ϑ* *λ*	61.3437 0.6155 21.9239	4.7070 0.2655 2.4844	64.912	-123.824	0.1510	0.2558
	EG-E	*a* *ϑ* *λ*	2.754×10^−1^ 1.314×10^3^ 5.371×10	5.645×10^−2^ 4.946 3.011	65.612	-125.224	0.1786	0.1131
	MO-E	*ϑ* *λ*	107.831 25.757	2.983 1.272	55.567	-107.135	0.2230	0.0227
	E	*λ*	5.0788	0.7564	28.128	-54.256	0.4949	5.318×10^−10^

The best model is chosen as the one having lowest AIC (Akaike Information Criterion). The results obtained in Table 7 indicated that the exponentiated generalized Marshall Olkin exponential distribution (EGMO-E) is a better distribution to model the death and recovery rate of covid 19 data than the other distributions.

**Fig 1:**
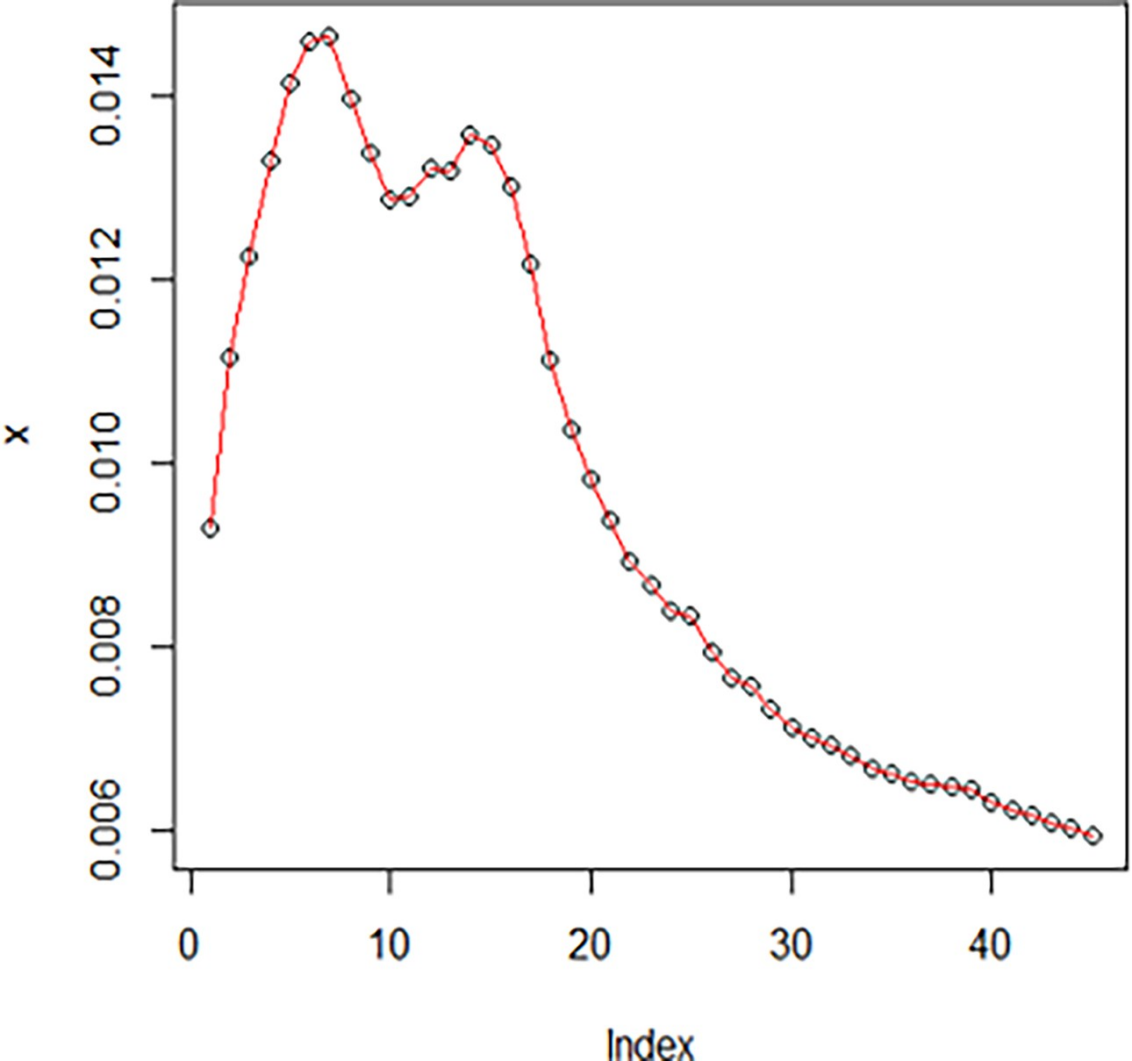
The death rate in Saudi Arabia from 1 April 2020 to 15 May 2020.

**Fig 2:**
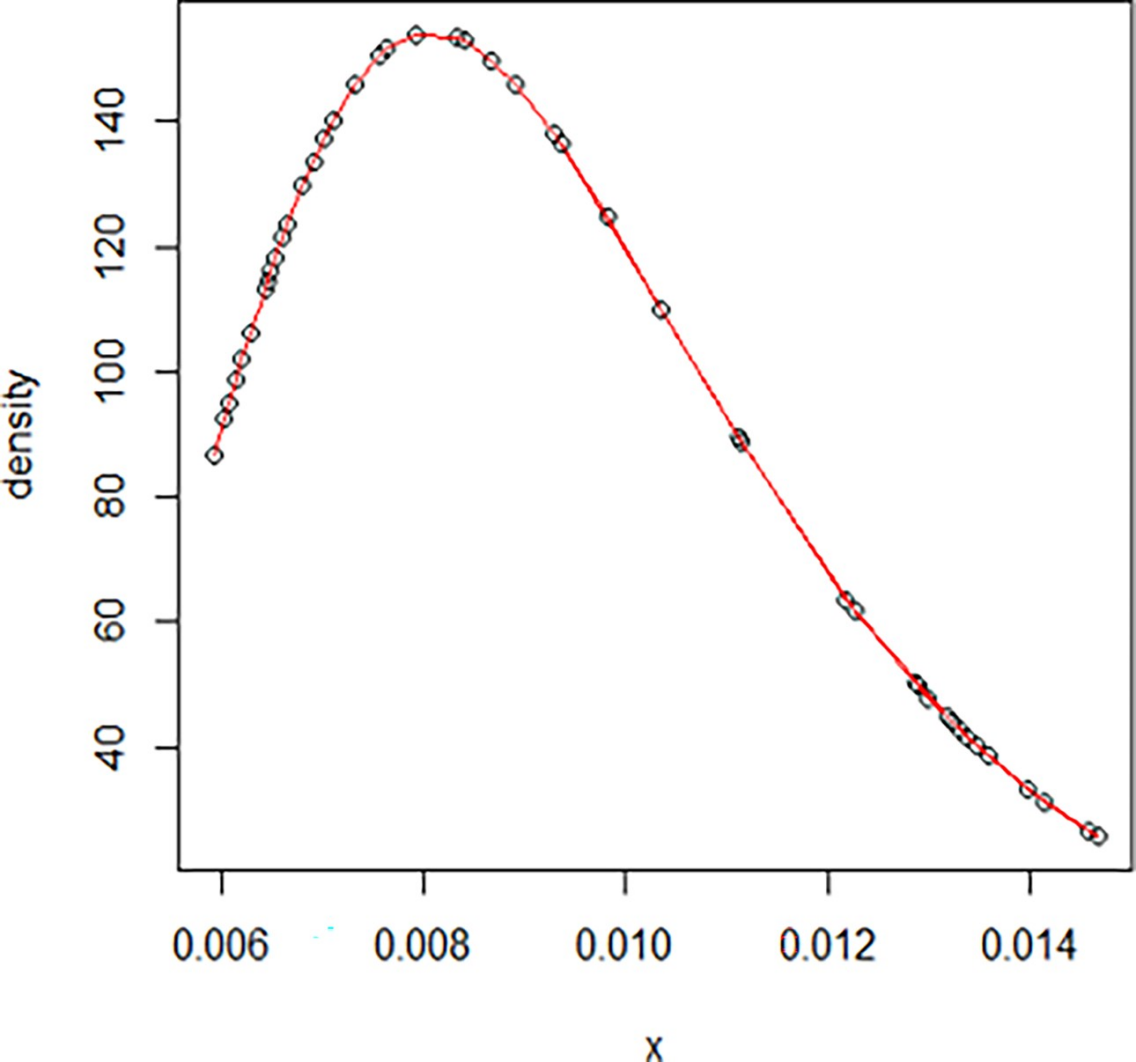
The estimated PDF of the death rate.

**Fig 3:**
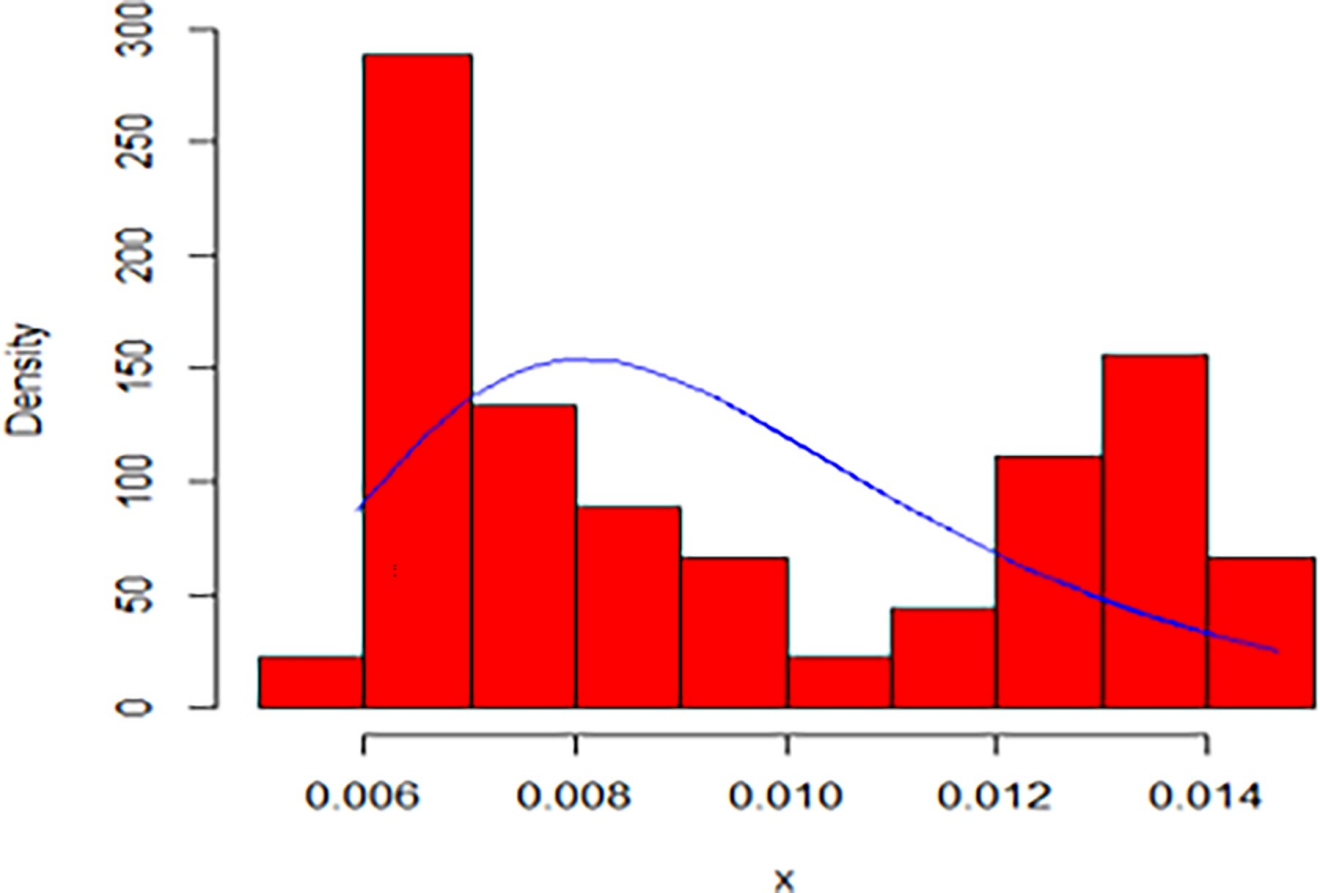
The estimated PDF with histogram of the death rate.

**Fig 4:**
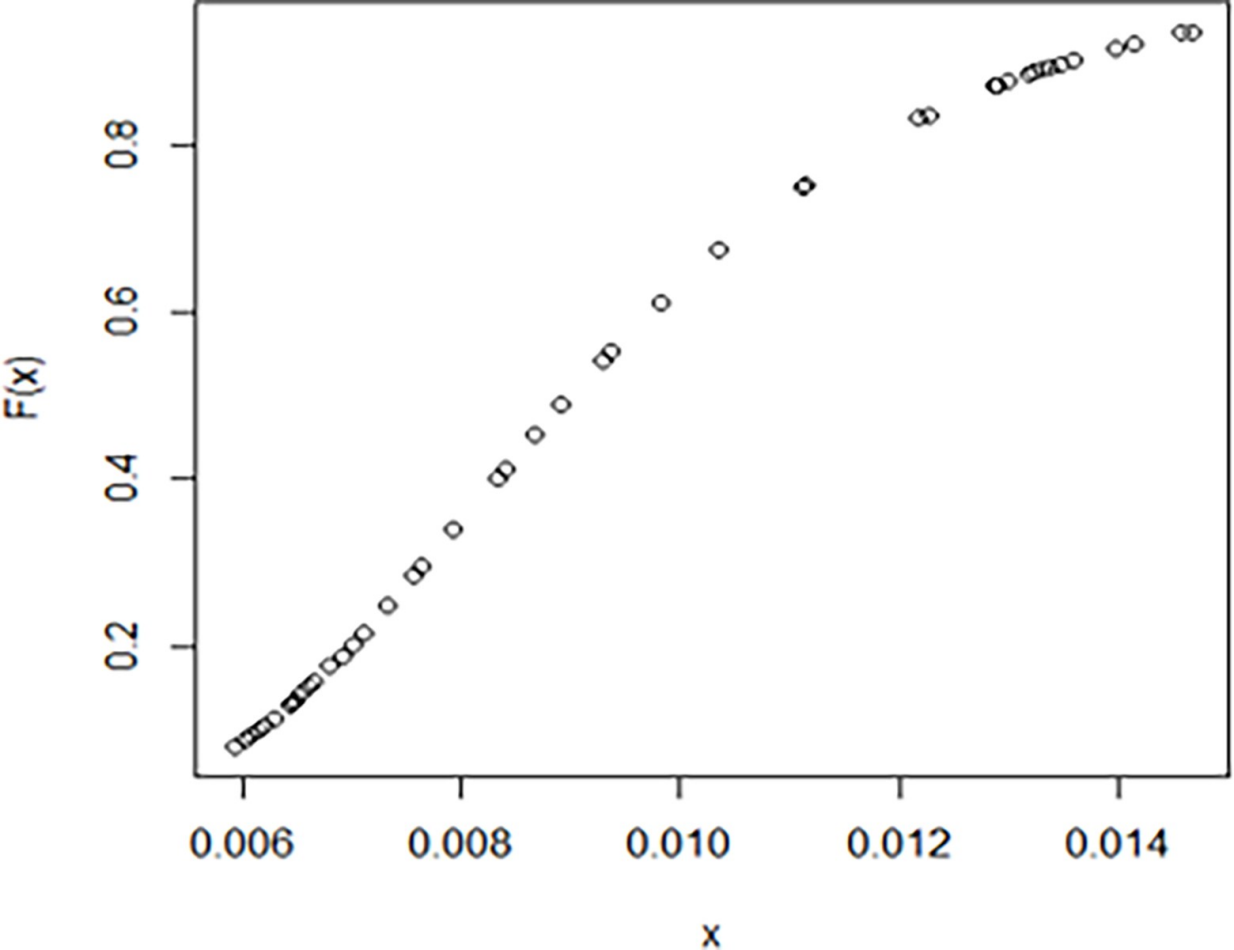
The estimated CDF of the death rate.

**Fig 5:**
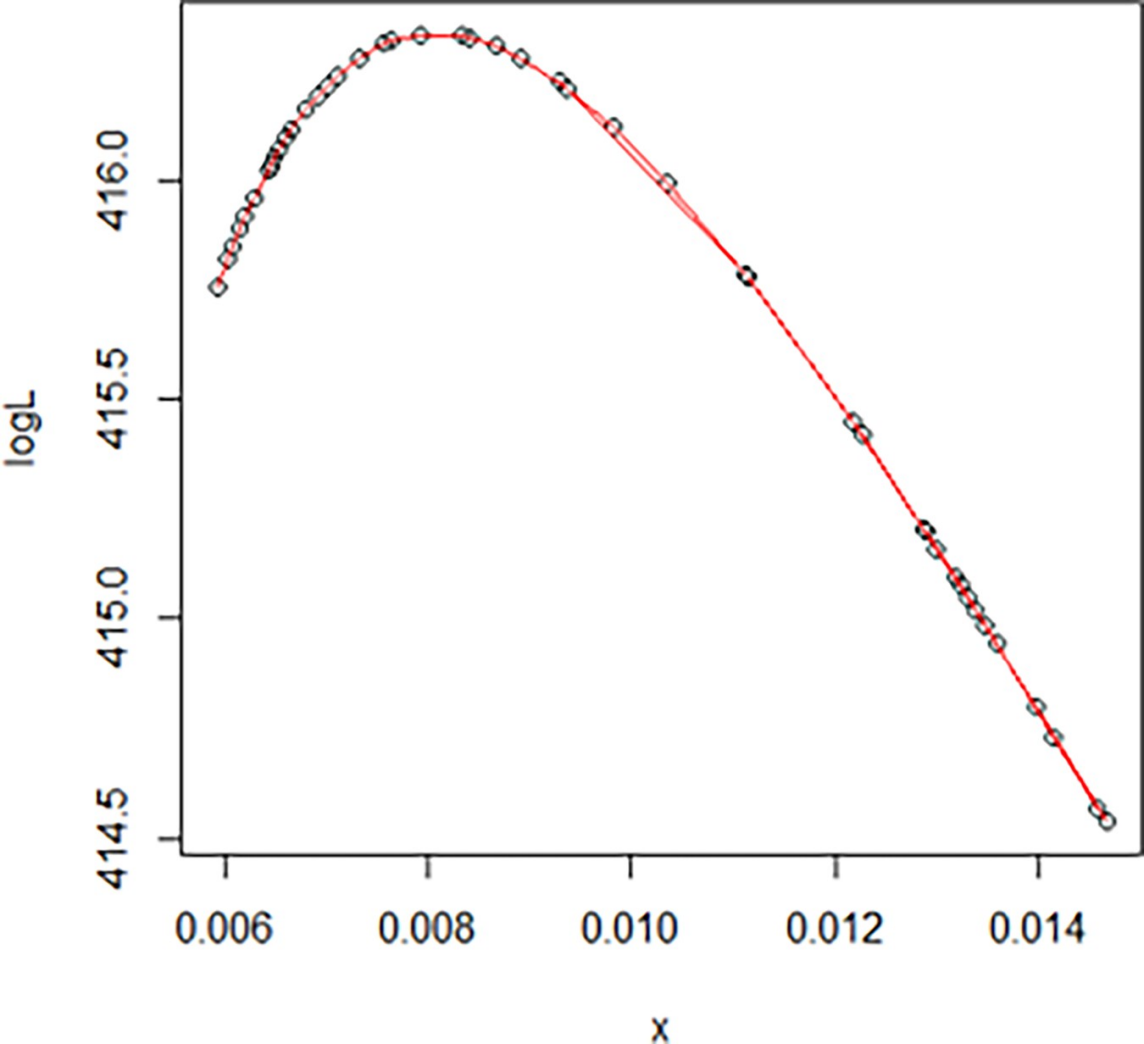
The log likelihood function of the death rate.

**Fig 6:**
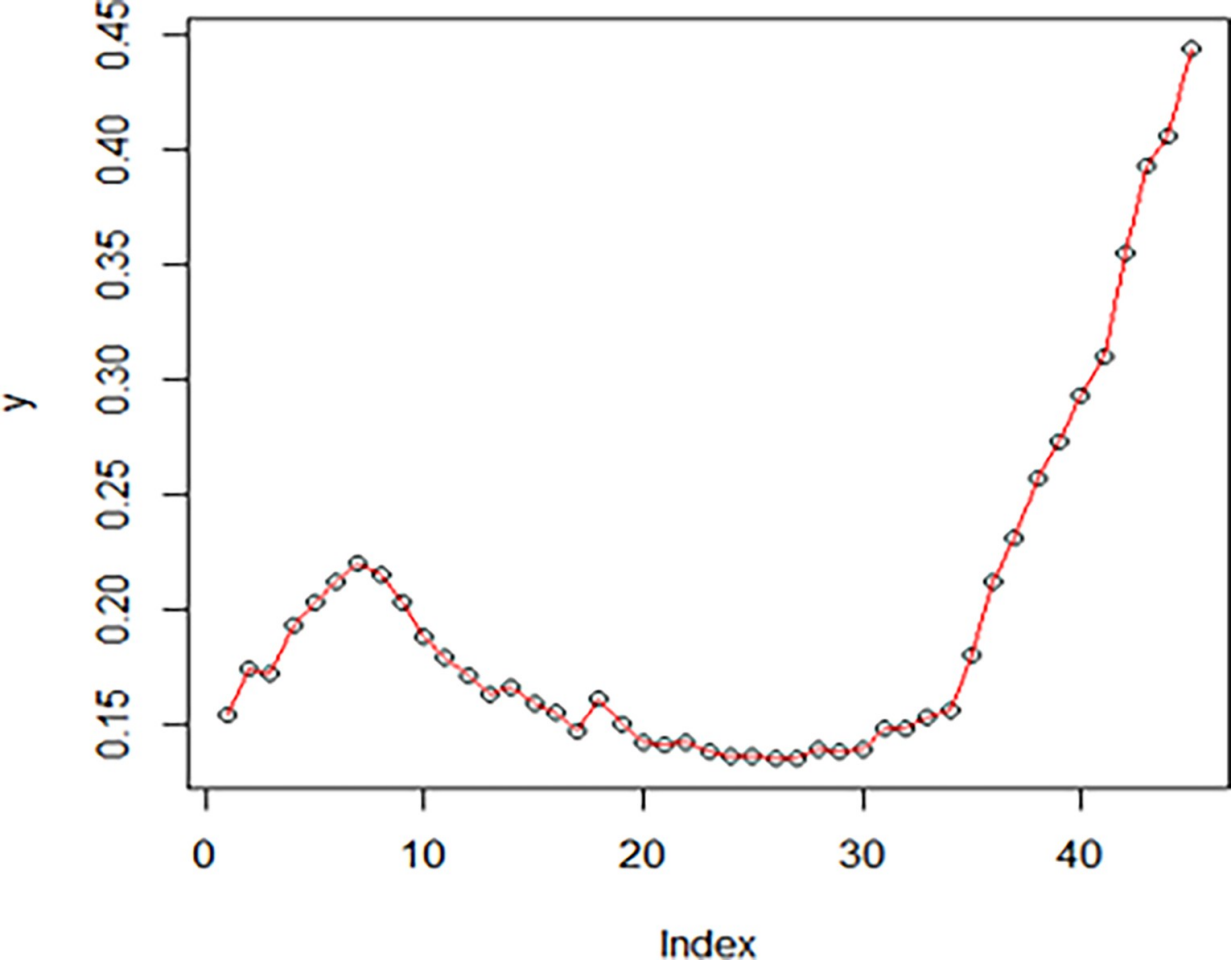
The recovery rate in Saudi Arabia from 1 April 2020 to 15 May 2020.

**Fig 7:**
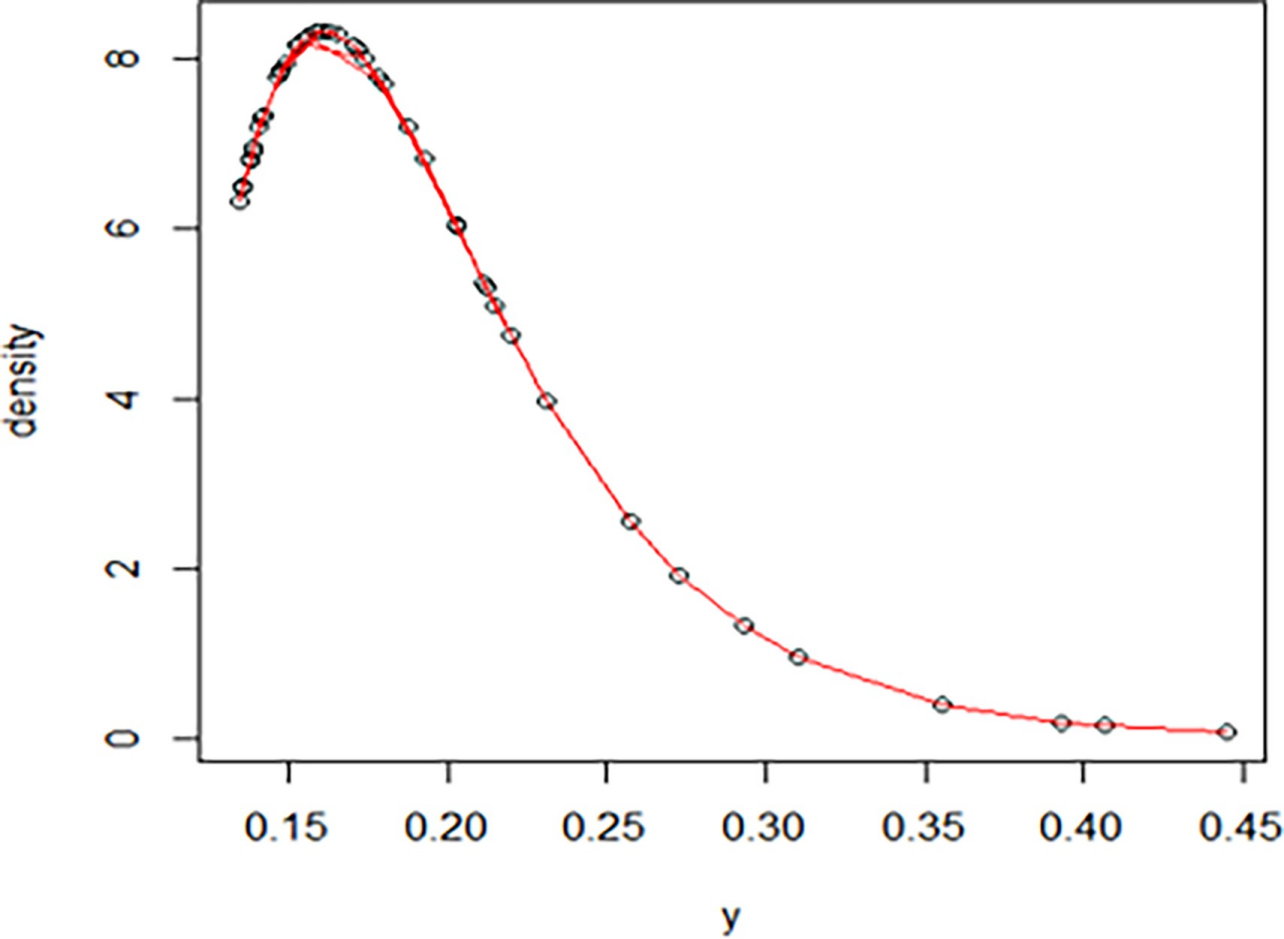
The estimated PDF of the recovery rate.

**Fig 8:**
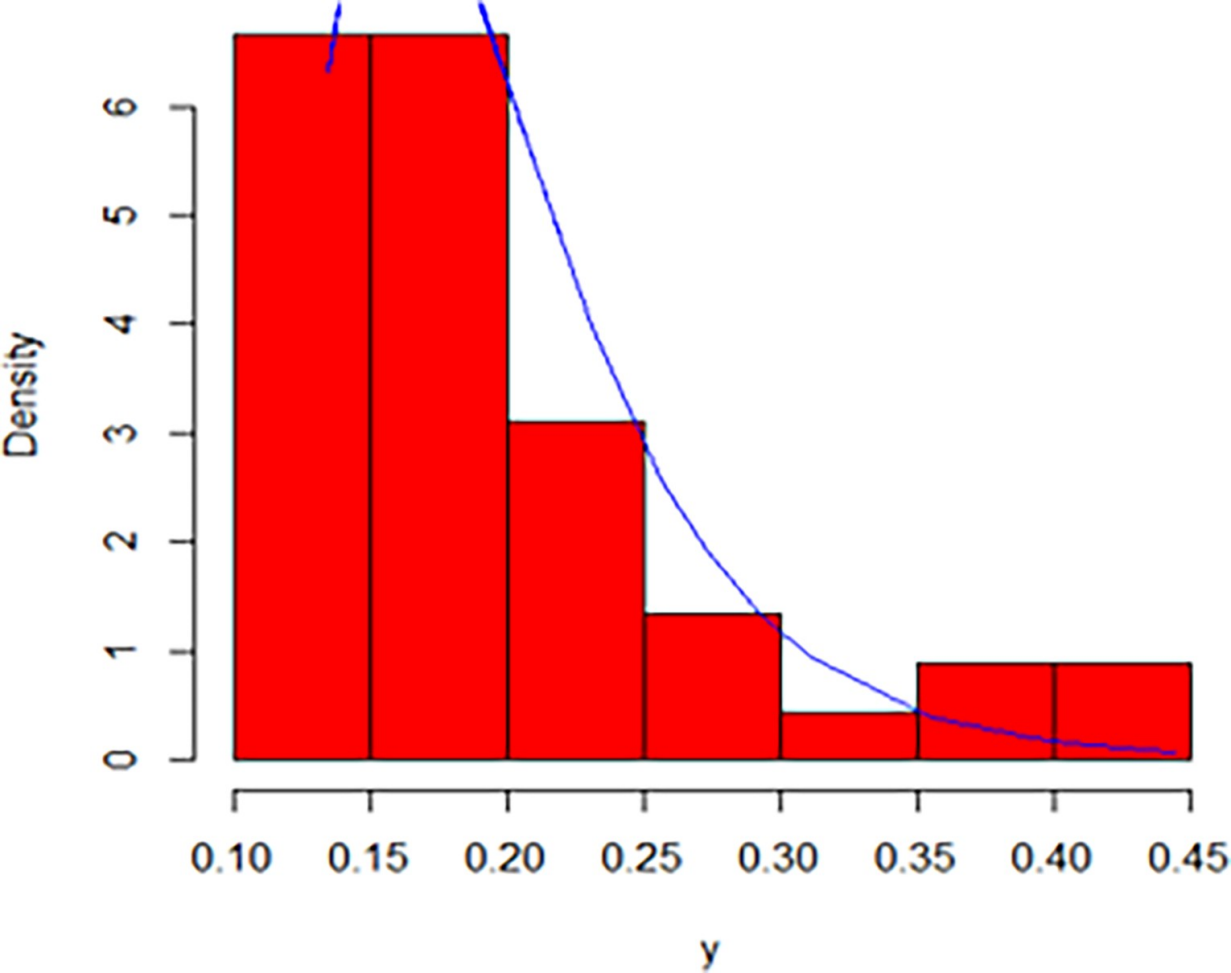
The estimated PDF with histogram of the recovery rate.

**Fig 9:**
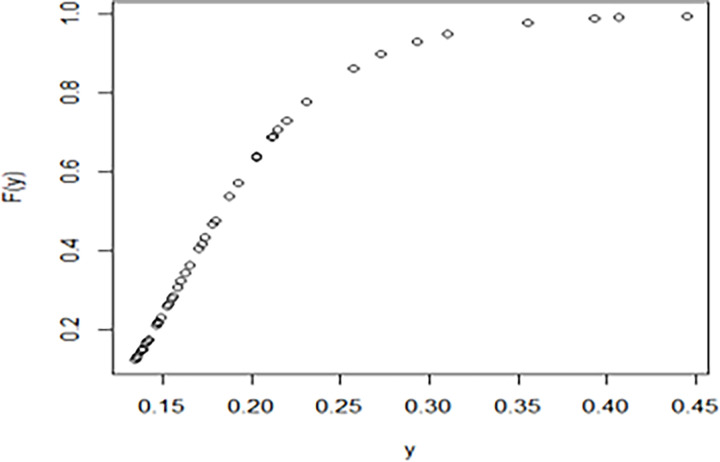
The estimated CDF of the recovery rate.

**Fig 10:**
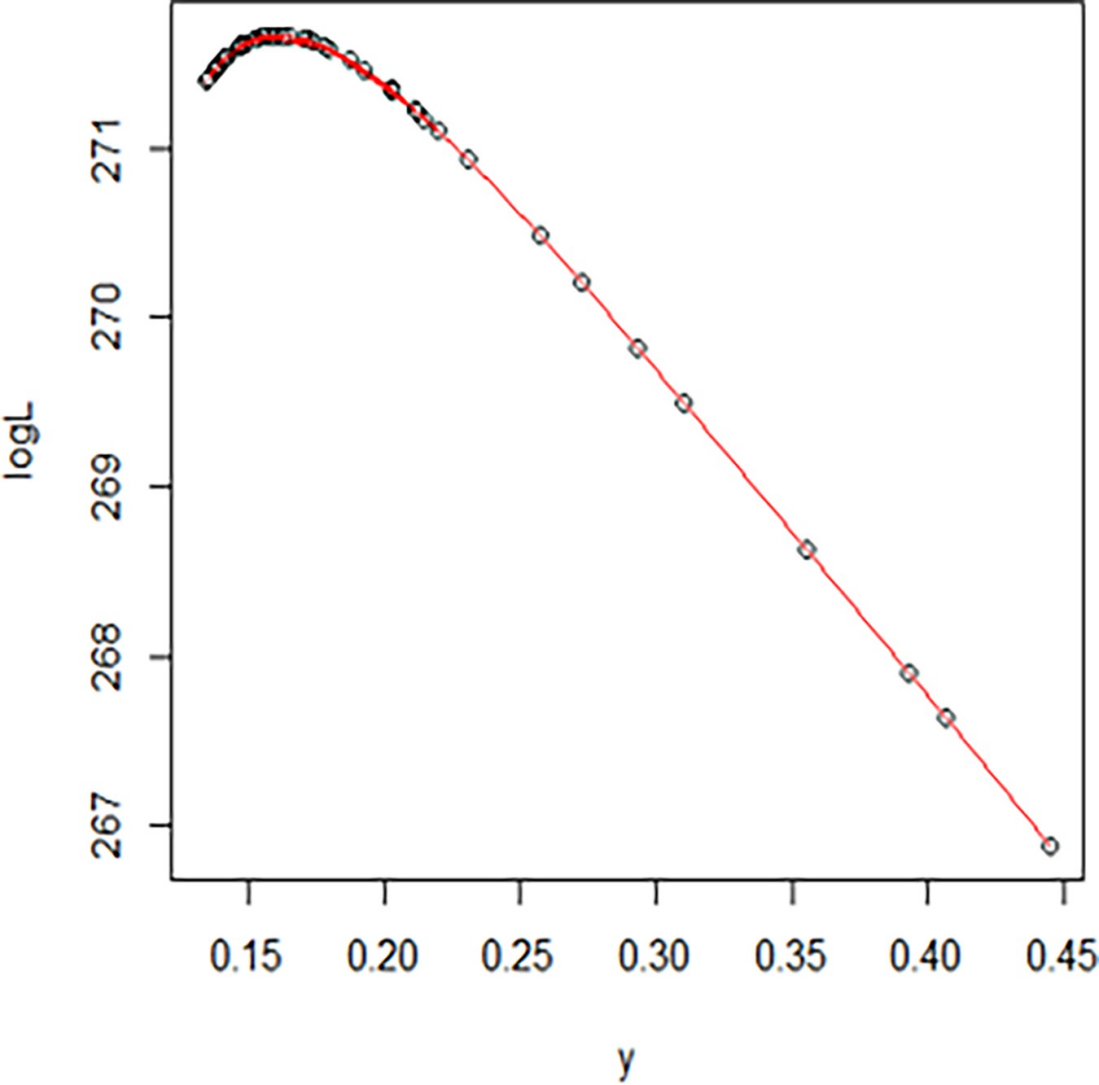
The log likelihood function of the recovery rate.

The maximum likelihood estimate of the reliability of the stress-strength model according to the death and recovery rate is obtained as 0.5175 and the 95% asymptotic confidence interval of R is [0.4143, 0.6206]. The Bayesian estimate of the reliability of the stress-strength model according to prior I is obtained as 0.8085 and the 95% credible confidence interval of R (with T = 1000) is [0.5810, 0.9603]. The Bayesian estimate of the reliability of the stress-strength model according to prior II is obtained as 0.7297 and the 95% credible confidence interval of R (with T = 1000) is [0.5802, 0.8560]. The death rate in Saudi Arabia from 1 April 2020 to 15 May 2020, the estimated PDF of the death rate, the estimated PDF with histogram of the death rate, the estimated CDF of the death rate and the log likelihood function of the death rate are presented in Figs 1–5, respectively. The recovery rate in Saudi Arabia from 1 April 2020 to 15 May 2020, the estimated PDF of the recovery rate, the estimated PDF with histogram of the recovery rate, the estimated CDF of the recovery rate and the log likelihood function of the recovery rate are presented in Figs 6–10, respectively.

## 10. Real data Application 2

A real data application is introduced to demonstrate how the proposed estimation techniques can be applied in practice. The following datasets were provided by Badar and Priest [21]. Here are the single fibers of 20 mm (dataset 1) and 10 mm (dataset 2) in gauge lengths.

Dataset 1 (n = 69): 0.312, 0.314, 0.479, 0.552, 0.700, 0.803, 0.861, 0.865, 0.944, 0.958, 0.966, 0.997, 1.006, 1.021, 1.027, 1.055, 1.063, 1.098, 1.140, 1.179, 1.224, 1.240, 1.253, 1.270, 1.272, 1.274, 1.301, 1.301, 1.359, 1.382, 1.382, 1.426, 1.434, 1.435, 1.478, 1.490, 1.511, 1.514, 1.535, 1.554, 1.566, 1.570, 1.586, 1.629, 1.633, 1.642, 1.648, 1.684, 1.697, 1.726, 1.770, 1.773, 1.800, 1.809, 1.818, 1.821, 1.848, 1.880, 1.954, 2.012, 2.067, 2.084, 2.090, 2.096, 2.128, 2.233, 2.433, 2.585, 2.585.

Dataset 2 (m = 63): 0.101, 0.332, 0.403, 0.428, 0.457, 0.550, 0.561, 0.596, 0.597, 0.645, 0.654, 0.674, 0.718, 0.722, 0.725, 0.732, 0.775, 0.814, 0.816, 0.818, 0.824, 0.859, 0.875, 0.938, 0.940, 1.056, 1.117, 1.128, 1.137, 1.137, 1.177, 1.196, 1.230, 1.325, 1.339, 1.345, 1.420, 1.423, 1.435, 1.443, 1.464, 1.472, 1.494, 1.532, 1.546, 1.577, 1.608, 1.635, 1.693, 1.701, 1.737, 1.754, 1.762, 1.828, 2.052, 2.071, 2.086, 2.171, 2.224, 2.227, 2.425, 2.595, 3.220.

The maximum likelihood estimates of the parameters for different distributions are calculated with their standard errors for the two datasets. Also, the log-likelihood function (Log L), Akaike information criteria, Bayesian information criteria and Kolmogorov–Smirnov (KS) test statistics are calculated for the two datasets. The goodness of fit for the exponentiated generalized Marshall Olkin Weibull distribution (EGMO-W) is compared with the exponentiated generalized Weibull distribution (EG-W), the generalized Marshall Olkin Weibull distribution (GMO-W), the exponentiated Marshall Olkin Weibull distribution (EMO-W), the generalized Weibull distribution (G-W), the Marshall Olkin Weibull distribution (MO-W) and Weibull distribution (W). The maximum likelihood estimators (MLE) for the parameters, Log L, AIC, BIC and KS for the dataset 1 and dataset 2 are shown in Tables 8 and 9, respectively.

**Table 8:** MLE estimates, Log L, AIC, BIC and KS for the data set 1.

Distribution	Parameters	Estimates	Standard error	Log L	AIC	BIC	KS	P-value
EGMO-W	*a* *b*_1_ *ϑ* *γ* *β*	1.279 0.409 0.161 6.842 2.470	0.263 0.060 0.018 0.329 0.063	-48.566	107.133	100.626	0.041	0.999
EG-W	*a* *b*_1_ *γ* *β*	1.532×10^−3^ 2.804×10^−1^ 7.758 9.076×10^−1^	3.559×10^−4^ 3.729×10^−2^ 2.125×10^−7^ 1.027×10^−4^	-52.152	112.304	107.798	0.106	0.419
GMO-W	*b*_1_ *ϑ* *γ* *β*	4.661×10^3^ 1.303×10^−3^ 1.006 5.826×10^−1^	2.166 7.508×10^−4^ 1.651×10^−1^ 2.014×10^−1^	-60.620	129.241	124.734	0.142	0.119
EMO-W	*a* *ϑ* *γ* *β*	6.231 0.106 12.500 5.700	0.300 0.000 0.458 0.059	-127.681	263.363	258.856	0.997	< 2.2e-16
G-W	*b*_1_ *γ* *β*	13.480 0.205 0.040	6.147 0.074 0.030	-161.996	329.993	327.486	0.740	< 2.2e-16
MO-W	*ϑ* *γ* *β*	4.902×10^4^ 2.025×10^−1^ 5.736×10^−5^	4.194 3.861×10^−2^ 1.238×10^−5^	-210.683	427.366	424.860	0.885	< 2.2e-16
W	*γ* *β*	0.184 0.013	0.017 0.003	-240.291	484.583	484.076	0.833	< 2.2e-16

**Table 9:** MLE estimates, Log L, AIC, BIC and KS for the data set 2.

Distribution	Parameters	Estimates	Standard error	Log L	AIC	BIC	KS	P-value
EGMO-W	*a* *b*_2_ *ϑ* *γ* *β*	0.013 0.483 19.574 2.783 0.379	0.033 0.374 3.485 1.777 0.179	-55.077	120.154	113.648	0.064	0.954
EG-W	*a* *b*_2_ *γ* *β*	6.418×10^−4^ 3.182×10^−1^ 4.188 3.769×10^−1^	2.770×10^−4^ 4.623×10^−2^ 1.231×10^−6^ 5.095×10^−2^	-62.337	132.674	128.168	0.142	0.152
GMO-W	*b*_2_ *ϑ* *γ* *β*	3.626×10^3^ 1.938×10^−3^ 6.943×10^−1^ 3.005×10^−1^	3.063 1.783×10^−3^ 1.821×10^−1^ 2.453×10^−1^	-62.940	133.882	129.374	0.116	0.359
EMO-W	*a* *ϑ* *γ* *β*	1.498 11.400 0.112 0.008	0.330 5.259 0.017 0.0009	-182.001	372.003	367.496	0.527	1.221e-15
G-W	*b*_2_ *γ* *β*	10.508 0.135 0.043	1.498 0.019 0.008	-198.150	402.300	399.794	0.853	< 2.2e-16
MO-W	*ϑ* *γ* *β*	3.016×10^2^ 2.532×10^−1^ 5.411×10^−2^	2.966 2.770×10^−2^ 1.654×10^−3^	-270.938	547.877	545.370	0.950	< 2.2e-16
W	*γ* *β*	0.183 0.011	0.018 0.003	-282.079	568.158	567.652	0.829	< 2.2e-16

The best model is chosen as the one having lowest AIC (Akaike Information Criterion) and BIC (Bayesian Information Criterion). The results obtained in Tables 8 and 9, indicated that the exponentiated generalized Marshall Olkin Weibull distribution (EGMO-W) is a better distribution to model the datasets than the other distributions.

**Fig 11:**
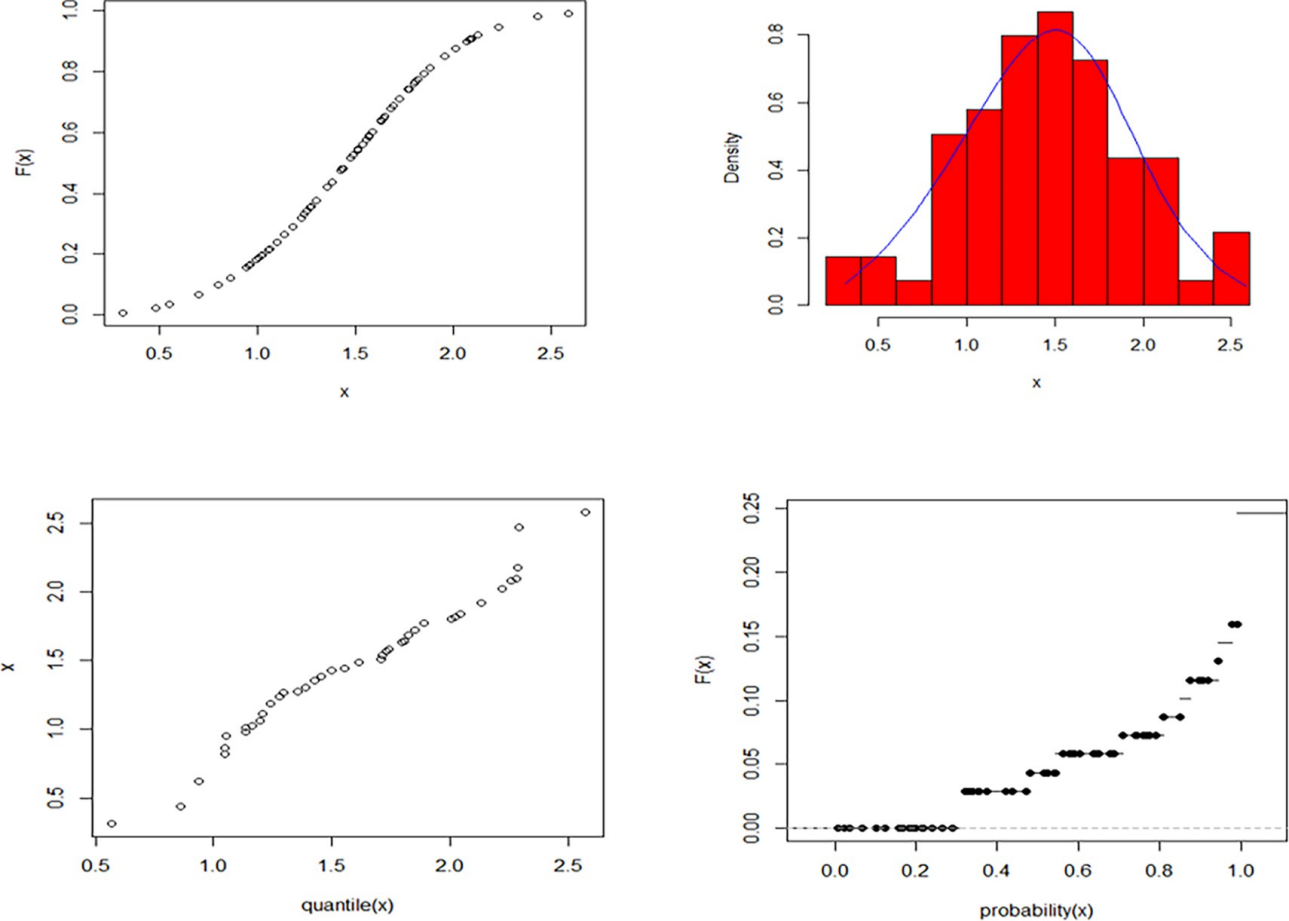
Estimated CDF, estimated PDF with histogram, Q-Q and P-P plots for EGMO-W distribution for dataset 1.

**Fig 12:**
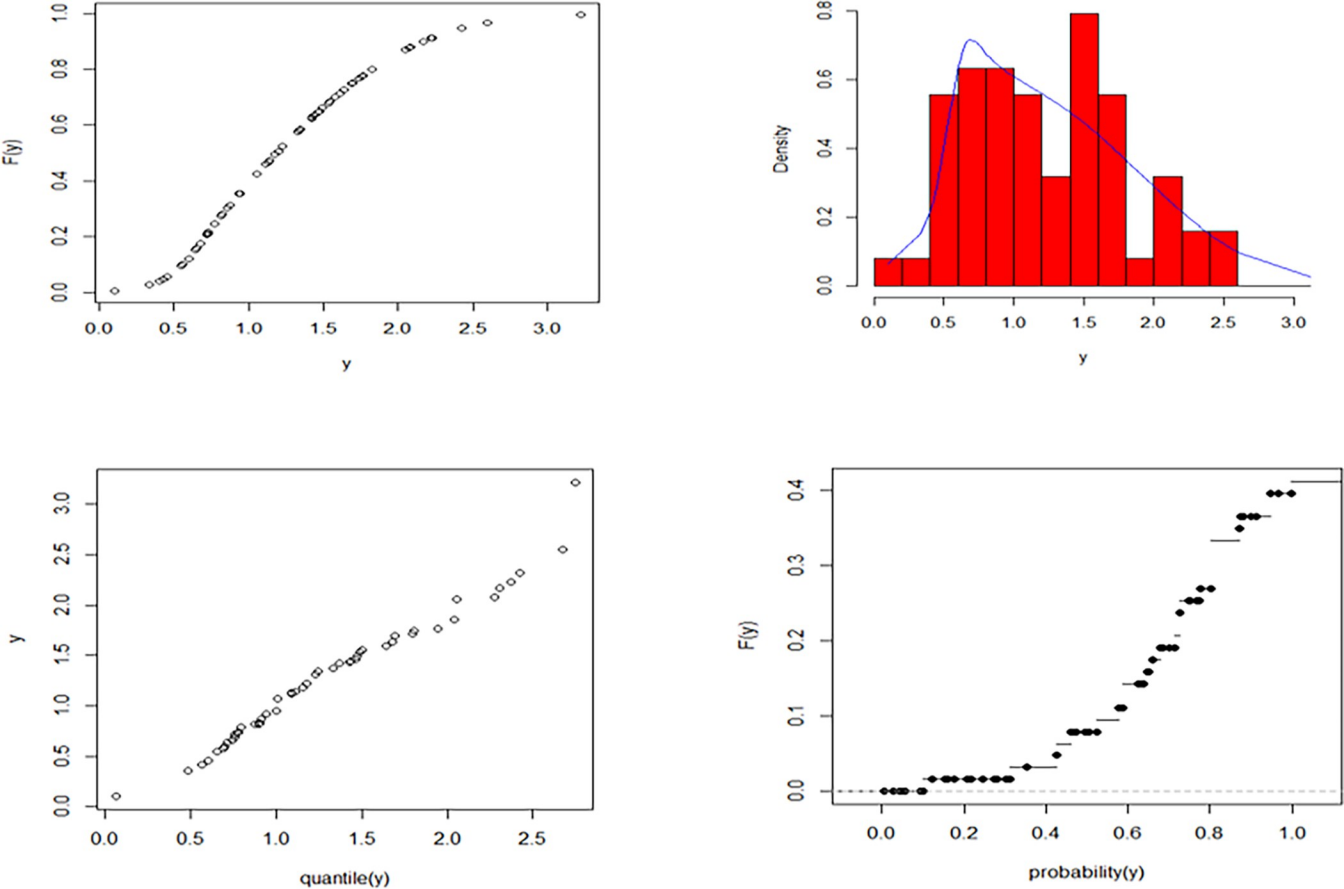
Estimated CDF, estimated PDF with histogram, Q-Q and P-P plots for EGMO-W distribution for dataset 2.

The maximum likelihood estimate of the reliability of the stress-strength model according to data set 1 and data set 2 is obtained as 0.458 and the 95% asymptotic confidence interval of R is [0.391, 0.524]. The non-informative priors (ξ_i_ = *η*_*i*_ = 0, *i* = 1,2,3,4) Bayesian estimate for the reliability of the stress-strength model according to dataset 1 and dataset 2 is obtained as 0.561 and the 90% credible confidence interval (with T = 10000) of R is [0.391, 0.534]. The informative priors (ξ_i_ = *η*_*i*_ = 0.5, *i* = 1,2,3,4) Bayesian estimate for the reliability of the stress-strength model according to data set 1 and data set 2 is obtained as 0.560 and the 90% credible confidence interval (with T = 10000) of R is [0.392, 0.533]. the estimated cumulative distribution function and empirical cumulative distribution function, estimated probability density function with histogram, Q-Q plot, and P-P plot for the EGMO-W distribution for dataset 1 and dataset 2 are shown graphically in Figs 11 and 12, respectively.

## 11. Conclusion

The study of the stress-strength reliability model subject to the exponentiated generalized Marshall Olkin G family of distributions is introduced. The maximum likelihood estimator, the asymptotic confidence and bootstrap confidence intervals for the stress-strength reliability function are obtained. Bayesian estimators and the credible interval for the stress-strength reliability function are derived. A simulation study is introduced in which EGMO-W distribution is applied. All results obtained in the simulation study is consistent. Applications based on real data are introduced to show the results for the stress-strength model and compare the EGMO-E and EGMO-W distributions with other different distributions. These comparisons show that EGMO-E and EGMO-W distributions can be considered as better models to fit the datasets.
